# The haplotype-phased genome assembly facilitated the deciphering of the bud dormancy-related QTLs in *Prunus mume*

**DOI:** 10.1093/dnares/dsae034

**Published:** 2024-12-04

**Authors:** Tzu-Fan Hsiang, Hisayo Yamane, Yuan-Jui Lin, Miku Sugimori, Soichiro Nishiyama, Kyoka Nagasaka, Ryohei Nakano, Ryutaro Tao

**Affiliations:** Graduate School of Agriculture, Kyoto University, Kyoto 606-8502, Japan; Graduate School of Agriculture, Kyoto University, Kyoto 606-8502, Japan; Graduate School of Agriculture, Kyoto University, Kyoto 606-8502, Japan; Graduate School of Agriculture, Kyoto University, Kyoto 606-8502, Japan; Graduate School of Agriculture, Kyoto University, Kyoto 606-8502, Japan; Experimental Farm, Graduate School of Agriculture, Kyoto University, Kyoto 619-0218, Japan; Experimental Farm, Graduate School of Agriculture, Kyoto University, Kyoto 619-0218, Japan; Graduate School of Agriculture, Kyoto University, Kyoto 606-8502, Japan

**Keywords:** *Prunus mume*, QTL, genome assembly, bud dormancy, chilling/heat requirement

## Abstract

Bud dormancy is a vital physiological process in woody perennials, facilitating their adaptation to seasonal environmental changes. Satisfying genotype-specific chilling requirements (CR) and heat requirements (HR) through exposure to specific chilling and warm temperatures is essential for dormancy release and the subsequent resumption of growth. The genetic mechanisms regulating bud dormancy traits in *Prunus mume* remain unclear. In this study, we first assembled the genome of ‘Nanko’, the leading *P. mume* cultivar in Japan, in a haplotype-resolved manner. Using an F_1_ segregating population from a cross between ‘Nanko’ (high-chill) and ‘SC’ (low-chill), a cultivar adapted to subtropical conditions, we identified quantitative trait loci (QTLs) for vegetative bud dormancy traits on chromosome 4 (LG4 QTLs) in the ‘Nanko’ genome and for CR and HR on chromosome 7 (LG7 QTL) in the ‘SC’ genome. A notable 5.6 Mb chromosome inversion was overlapped with LG4 QTL interval in one of the ‘Nanko’ haplotypes. We also identified candidate genes based on haplotyping, differential expression between the parents or the presence of trait-correlated variants in coding regions. Notably, genes such as *PmuMAIN*, *PmuNAC2*, *PmuDOG1*, *PmuSUI1*, *PmuATG8CL*, *PmubZIP44*, and *PmuSAUR50* were identified. This study provides valuable insights into the genetic regulation of vegetative bud dormancy in *Prunus* species.

## 1. Introduction

Bud dormancy, defined by the inability of buds to resume active growth, is a biological adaptation of deciduous fruit trees to seasonal environmental changes. Lang et al. (1987) classified dormancy into 3 categories: paradormancy, endodormancy, and ecodormancy. During paradormancy, bud growth is inhibited by distal organs, primarily due to apical dominance. As autumn approaches, with decreasing temperatures and shorter photoperiods, endodormancy is induced, where growth inhibition is governed by internal signals within the buds. To transition to the subsequent stage, ecodormancy, endodormant buds must be exposed to chilling temperatures for a specific duration, known as the chilling requirement. Ecodormancy is characterized by growth inhibition due to unfavourable environmental conditions.^1,2^ The buds’ heat requirement, which is the specific period of exposure to warm temperatures, must be met before blooming and leafing can occur.^3,4^ Consequently, temperature is a critical factor influencing the transitions between dormant stages.

*Prunus mume* Sieb. et Zucc. is a temperate deciduous fruit tree species belonging to the Rosaceae family, originating from Tibet, China.^5^*Prunus mume* has been extensively cultivated in East Asian countries including Taiwan, Korea, and Japan, serving both as a fruit and ornamental resource. There is considerable variation in chilling requirements (CR) and heat requirements (HR) among *P. mume* cultivars, leading to diverse blooming and leafing characteristics.^6–8^ However, the genetic regulation of the bud dormancy mechanism in *P. mume* remains unclear. To enhance our understanding of the genetic mechanisms underlying bud dormancy, bud break and blooming, quantitative trait loci (QTL) analyses have been conducted in various fruit tree species of *Prunus*, including sweet cherry (*P. avium*), peach (*P. persica*), apricot (*P. armeniaca*), almond (*P. dulcis*), and Japanese plum (*P. salicina*)^9–18^ (Tables S1 and S2). Due to the high homology among *Prunus* species genomes, QTLs for related traits were identified in similar chromosomal regions^11^; these QTLs exhibited high broad-sense heritability of quantitative traits in both floral and vegetative buds.^11,13,15,17^

Putative candidate genes located within QTL intervals on linkage group 1 (LG1) and linkage group 4 (LG4) controlling bud dormancy have already been proposed.^10,18–20^ In peach, the LG1 QTL interval contains *DORMANCY-ASSOCIATED MADS-box* (*DAM*) genes.^12,19^ In *Prunus*, *DAM* genes are linked to increased CR, delayed bud break, and growth inhibition.^18,20–24^ In sweet cherry, *DAM5* and *DAM6* are highly probable candidate genes within the LG1 QTL, associated with the regulation of the blooming date (BD) and CR.^10,25^ Conversely, studies on flower bud dormancy-associated LG4 QTL in sweet cherry suggest the likelihood of *EMBRYONIC FLOWER2* (*EMF2*), *ACTIN-RELATED PROTEIN 4* (*ARP4*), *BOI-RELATED E3 UBIQUITIN-PROTEIN LIGASE 3* (*BOI-E3*), *SERINE/ARGININE-RICH-LIKE PROTEIN 45A* (*SR45a*), and *SMALL AUXIN UP RNA71* (*SAUR71*) being potential candidates.^10,20^ Among them, SAUR71 is associated with the auxin response and is related to general growth and developmental regulation.^26,27^ We previously reported that vegetative bud dormancy-related QTL was localized on LG4 in 2 *P. mume* F_1_ segregating populations derived from ‘Nanko’ crossed with ‘SC’ (NKSC) and ‘Niao-ume’ (Ellching) crossed with ‘Nanko’ (NINK).^13^ While QTLs associated with vegetative bud dormancy in *P. mume* and flower bud dormancy in sweet cherry are both located on LG4, they occupy distinct genetic loci. Furthermore, our preceding research concurrently identified a QTL linked to the CR of vegetative buds (CRL) within LG7, suggesting that multiple regulatory mechanisms may underlie bud dormancy and bud break regulations in *Prunus*.^10,13^

Recent genomic studies of *P. mume* suggest that it has frequently acquired advantageous genes from other species in the subgenus *Prunus* during evolution.^28,29^ For example, introgression signals accompanied by selective sweep peaks in chromosome 2, 3, and 4 are unique in Japanese cultivars, independently of Chinese and Taiwanese cultivars.^28^ The population structure analysis and dissemination route of *P. mume* both showed that Taiwanese cultivars, Japanese cultivars, and Chinese cultivars were classified into different clusters.^29^ Although the genomic structure of Chinese cultivars may be distinct from Japanese cultivars,^28,30^ only *P. mume* V1.0 genome, which is used in the previous QTL study^13^ and assembled using a wild accession from Tibet, China,^5^ has been publicly available.

The aim of this study is to decipher the bud dormancy-related QTLs of *P. mume*. To achieve this, we first constructed a physical genome of *P. mume* ‘Nanko’, the most extensively grown cultivar in Japan, using 10X Genomics Chromium-Linked Read and PacBio HiFi Long-Read sequencing technologies. We then assembled the sequences and obtained haplotype-phased genome, enabling us to minimize the most probable QTL regions in LG4 and LG7 and identify candidate genes controlling vegetative bud dormancy.^13^

## 2. Materials and methods

### 2.1. Plant materials

Adult trees of *P. mume* ‘Nanko’, ‘SC’, and the F_1_ population derived from a cross between ‘Nanko’ and ‘SC’ pollen (NKSC F_1_ population)^13^ were utilized. These plants were grown at the Kizu experimental farm of Kyoto University, Japan. Leaves collected during the 2019–2020 and 2020–2021 seasons were immediately frozen in liquid nitrogen and subsequently stored at −80°C for DNA extraction. Dormant buds of ‘SC’ collected on October 25th, November 26th, December 25th, and January 24th during the 2019–2020 season were frozen in liquid nitrogen and stored at −80°C for RNA extraction.

### 2.2. Long-reads sequencing and genome assembly

High molecular weight genomic DNA (HMW gDNA) from young leaves of *P. mume* ‘Nanko’ was extracted using a modified cetyl-trimethylammonium bromide (CTAB) method, as adapted from previous studies.^31–33^ The concentration and purity of the HMW gDNA were evaluated using a Nanodrop 2000 spectrophotometer (Thermo Fisher Scientific Inc.) and a Qubit 4 fluorometer (Thermo Fisher Scientific Inc.). In addition, the integrity and quality of the HMW gDNA were assessed by pulsed-field gel electrophoresis (PFGE), performed by the BGI company, Beijing, China.

For genome assembly, 2 long-read sequencing technologies were employed: Circular Consensus Sequencing (CCS) (HiFi) from Pacific Biosciences and Linked Read Sequencing (10X Genomics Chromium). Initially, long-read libraries were sequenced using the CCS mode using 2 single-molecule, real-time (SMRT) cells on the PacBio Sequel II platform (Menlo Park, CA, USA). The libraries were converted into polymerase read output files on the instrument, which were then transformed into subreads after basic filtering using the SMRT Link V11.1 software. These subreads were subsequently converted into CCS reads using the CCS software.^34^ The SMRT sequencing of ‘Nanko’ HMW gDNA under CCS mode on the Pacbio Sequel II platform yielded a total of 329.596 Gb (~1390.114 × coverage) of polymerase reads. After processing with CCS software and filtered by a quality value of Q20, a total of 21.767 Gb (~91.807 × coverage) of HiFi reads were obtained. The mean read lengths from 2 cells were 15.833 kb and 15.881 kb, respectively (Table S3). HiFi reads were extracted from the CCS reads with quality values greater than or equal to Q20. The PacBio HiFi reads were de novo assembled into contigs using Hifiasm v. 0.16.1 (parameter: -D 20), generating assemblies of 2 haploids.^35^ To join the contigs, ntLink v1.1.2 (parameter: *k* = 32, *w* = 250) was employed.^36^ In addition, 10X Chromium linked reads were utilized to scaffold the contigs using arcs v. 1.0.6 software (parameter: *z* = 5000).^37^

Genotyping-by-sequencing (GBS) data of *P. mume* F_1_ populations (‘NKSC’ and ‘NINK’),^13^ derived from crosses of ‘Nanko’ and ‘SC’ pollen, and ‘Niao-ume’ (Ellching) with ‘Nanko’ pollen, respectively, were used for scaffolding. The 4 parental genetic maps for each haploid were constructed using JoinMap v. 4.1.^38^ Subsequently, ALLMAPS v. 1.2.7 was employed to integrate the information from these genetic maps for scaffolding.^39^ To address gaps in the assemblies, HiFi reads were applied with LR_Gapcloser v. 1.1 (parameter: -s p, -v 5000).^40^ The assembly results were assessed and refined using HiFi reads through Inspector.^41^

To further refine the diploid genome assembly and eliminate redundant sequences, HiFi reads, 10X Chromium linked reads, and BUSCO single copy read depths were utilized for correction with Diploidocus v. 1.1.4 (parameter: -runmode dipcycle).^42^ The pseudo-chromosomes of the diploid genome assembly were categorized into 2 haploids based on their sizes, with larger ones assigned to haploid 1 and smaller ones to haploid 2 for each pseudo-chromosome. The scaffolds were then sorted based on size. The quality and completeness of genome assemblies were assessed using Benchmarking Universal Single-Copy Orthologs (BUSCO) v. 4 with the embryophyta_odb10 dataset.^43^

### 2.3. Genome annotation

The annotation process was carried out on GenSAS v. 6.0 platform.^44^ Interspersed repeated sequences and low complexity DNA sequences of the ‘Nanko’ diploid genome assembly were identified using RepeatMasker v. 4.1.1, utilizing the search engine of NCBI and the DNA resource of plnrep.ref (parameter: repeatmasker_speed_sel: -q) (https://www.repeatmasker.org/) and used for annotation.

RNA-seq datasets of various tissues (fruit, stem, root, leaf, and flower bud) of *P. mume* (NCBI BioProject: PRJNA172987),^5^ and ‘Nanko’ vegetative buds at different stages (NCBI BioProject: PRJNA958110)^45^ were aligned to the ‘Nanko’ diploid genome assembly using blastn, BLAT, HISAT2, PASA, and TopHat for transcript annotation.^46–50^ The resulting Binary Alignment/Map (BAM) files and the soft-masked sequence were used for structural annotation with BRAKER2 v. 2.1.5.^51^ The gene set obtained was refined using the transcript sequence of the *P. mume* V1.0 genome through PASA v. 2.4.1.^5,49^ For functional annotations, the protein sequences from the *P. mume* ‘Nanko’ diploid assembly were annotated using the Swiss-prot and eggNOG databases.^52–55^

### 2.4. Comparative genomic analyses

Genomic synteny analyses were conducted using Minimap2, comparing the 2 haploids of ‘Nanko’ genomes, the *P. mume* V1.0 genome, and other *Prunus* species genomes on the D-GENIES platform.^56–61^ The species tree analysis was conducted with ORTHOFINDER using default settings,^62^ incorporating *P. mume* ‘Nanko’ and 14 other species.^5,57–61,63–70^ The One-Step MCScanX program within Tbtools^71,72^ was utilized to analyse synteny between the ‘Nanko’ haploid 1 and 2 assemblies. Gene density and variant distribution [snp (single nucleotide polymorphism), sindel (small insertion/deletion), DEL (deletion), INS (insertion), and INV (inversion)] were visualized using the Advanced Circos feature of Tbtools.^72^

### 2.5. Phenotyping of bud dormancy-related phenotypes in the ‘NKSC’ F_1_ population

During the 2021–2022 season, hourly temperature data were recorded from 6 October 2021, until 16 February 2022, when all individuals had reached their leafing dates, using a Thermo Recorder TR-51i data logger (T&D Corporation, Nagano, Japan) (Fig. S1). *P. mume* ‘Nanko’ (high-chill), ‘SC’ (low-chill), and 60 individuals from the F_1_ progeny, ‘NKSC’ population at the Kyoto University Experimental Farm in Kyoto, Japan, were utilized for this study. Shoots were collected on October 28th, November 11th and 25th, December 9th,16th, 23rd, and 29th, 2021, as well as January 6th, 13th, 20th, and 27th, 2022. The basal portions of these shoots were immersed in water containing 1% (v/v) Misakifarm, a Japanese floral preservative designed to extend the vase life of cut flowers, and subjected to forcing conditions (23°C, 16 h/ 8 h photoperiod) inside a Versatile Environmental Test Chamber MLR-351 (Sanyo, Osaka, Japan). Phenotypic observations were made once every 1–4 days. For the bud break competency test, vegetative buds were evaluated after 21 days. The fulfilment of chilling requirement was defined at the sampling date when 50% of leaf buds reached at BBCH stage 03 in forcing condition (Table S4). The chill hours (CH) model was used to calculate CR.^73^ Leafing was evaluated in the field as the date when 50% of leaf buds reached BBCH stage 10 (Table S4). The date when more than 3 designated shoots met the above-described criteria was regarded as the leafing date (LD), which was recorded as the number of days after the first individual started leafing.

For HR calculation, 2 methods were employed: the leafing time (LT) method (2020−2021 season) and growing degree hour (GDH) method (2013−2014, 2014−2015, and 2021−2022 seasons).^13,74^ For the LT method, bud break timing was initially defined as the time points when 40% of vegetative buds in 3 out of 5 shoots reached BBCH stage 10 in forcing condition (23°C, 16 h/ 8 h photoperiod) (Table S4). The number of hours accumulated under the forcing condition until bud break timing was considered as HR. Shoots were collected on 20 January 2021. For the GDH method,^75^ the sum of hourly average temperatures minus the base temperature (4.5°C) (temperatures over 25°C were considered as 25°C) was calculated after the fulfilment of CR until LD in field. The records of CR, LD and hourly temperature data from the 2013−2014 and 2014−2015 seasons were adopted from the previous study.^13^

### 2.6. Identification of vegetative bud dormancy-associated QTLs

A dataset comprising GBS information from 101 individuals of the NKSC F_1_ population and their parents was used.^13^ Fastp was used to assess the quality of all sequencing data, perform adapter trimming, and conduct quality filtering.^76^ Clean reads were then mapped to the ‘Nanko’ haplotype 1 genome using Burrow-Wheeler Aligner (BWA) v. 0.7.17.^77^ The resulting BAM files were processed using SAMtools v. 1.10 mpileup option, and variants were called using BCFtools v. 1.9.^78^ The variants were filtered, and indels were removed using VCFtools v. 0.1.17 with the parameters: --max-alleles 2 --min-alleles 2 --minQ 10 --minDP 10 --maf 0.05 --max-missing 0.7 --remove-indels.^79^ SNP genotypes corresponding to ‘’lm × ll” (heterozygous in ‘Nanko’), ‘hk × hk’ (heterozygous in both parents), ‘nn × np’ (heterozygous in ‘SC’) were used to construct the parental genetic maps using JoinMap v. 4.1 with default setting.^38,80^ SNP loci not relevant to linkage mapping were excluded based on the following criteria: (i) missing in over 20% of individuals; (ii) exhibiting significant segregation distortion based on a χ^2^ test (*P* < 0.01); and (iii) identical or highly similar (> 95% similarity). Individuals with fewer than half of the markers were removed. In addition, any remaining markers that were highly similar (>95%) were excluded. QTLs were detected using the interval mapping method in MapQTL v. 6.0.^38^ QTLs with significant logarithm of odds (LOD) scores (*P* < 0.05) were identified. The confidence interval of each QTL was determined as 1 and 1.5-LOD intervals from its peak, with the LOD above the threshold of 3 and the permutation test threshold (*P* < 0.05 based on 1000 replicates). The linkage maps with QTL intervals were plotted using MapChart.^81^

### 2.7. Haplotype analysis within QTL intervals

A whole-genome re-sequencing dataset was generated from 60 NKSC F_1_ individuals. DNA libraries were constructed using the DNBSEQ-T10×4RS High-throughput Sequencing Set (FCL PE150) and sequenced on the DNBSEQ-T10×4RS sequencer, achieving coverage of over 30× per sample. Low-quality reads were filtered out using Fastp.^76^ The clean reads were aligned to the *P. mume* ‘Nanko’ haplotype 1 genome with bwa (v. 0.7.17), employing BWA-MEM algorithm with default settings. The 8 pseudo-chromosomes of the ‘Nanko’ haploid 1 assembly served as the reference genome. Duplicate reads were removed using Picard Toolkit’s MarkDuplicates tool (GitHub Repository. https://broadinstitute.github.io/picard/). The resulting BAM files were processed using SAMtools v. 1.10 for piling-up, and variants were called using BCFtools v. 1.9.^78^ The variants were filtered using VCFtools v. 0.1.17 using parameters: --max-alleles 2 --min-alleles 2 --minQ 20 --minDP 20 --maf 0.05 --max-missing 0.9.^79^ SNP and sindel variants were separately extracted using VCFtools v. 0.1.17 with the --remove-indels or --keep-only-indels options. Missing genotypes were imputed using Beagle 5.0 with default settings (‘Imputed_snp_set’ and ‘Imputed_sindel_set’).^82,83^ For haplotype analysis, the ‘imputed_snp_set’ VCF file is merged with the GBS VCF file containing SNPs from an additional 41 individuals, to be used in subsequent haplotype analysis.

‘Nanko’ and ‘SC’ were the donor parents for LG4 and LG7 QTLs controlling vegetative bud dormancy traits, respectively.^13^ Thus, haplotype analyses were conducted using the geneHapR package (version 1.1.9), utilizing SNPs with ‘lm × ll’ (heterozygous in ‘Nanko’; homozygous in ‘SC’) or ‘nn × np’ (homozygous in ‘Nanko’; heterozygous in ‘SC’) for LG4 and LG7 QTL, respectively. The trait-haplotype associations were visualized using the ggplot2 package.^84,85^ Statistical analyses were conducted using Student’s *t*-test and the Tukey honestly significant difference test in R Studio, with a significance threshold of *P*-value < 0.05 to identify statistically significant differences.

### 2.8. Identification of loss-of-function structural variants within QTL intervals

The BAM files from the re-sequencing data of 60 NKSC F_1_ offspring and their parents were utilized. Structural variants (SVs) were identified across the population samples using DELLY2 v.1.1.8.^86^ SVs that passed the DELLY’s quality filters (flag PASS) were retrained. Subsequently, variants (DEL, INV, and INS) were extracted to form the ‘Delly_SV_set’. Variants (snp, sindel, DEL, INV, and INS) within the LG4 and LG7 QTL intervals were filtered based on their strong correlation with bud dormancy-associated phenotypes (|*r*|≧0.6), and determined via the Pearson correlation coefficient using the R package ‘corrplot’ with VCF files (‘Imputed_snp_set’, ‘Imputed_sindel_set’, and ‘Delly_SV_set’) (GitHub Repository: https://github.com/taiyun/corrplot). Variants located within coding sequence (CDS) were then extracted using BEDTools.^87^ For the LG4 QTL, variants with ‘lm × ll’ or ‘hk x hk’ genotypes were used for the analysis, while for the LG7 QTL, variants with ‘nn × np’ or ‘hk x hk’ genotypes were used.

### 2.9. Detection of chromosomal inversion using PCR

The primer set Pmu4_1_F (5ʹ-CCTCTTATACCTTTGGCGATGGAC-3ʹ, forward) and either Pmu4_1_R1 (5ʹ-CTAACCCTTCTTGTCCCAATGTGC-3ʹ, reverse) or Pmu4_1_R2 (5ʹ-GGATACCATGTTGAGGGAATGAGC-3ʹ, reverse) was used for PCR amplification to detect chromosomal inversion on chromosome 4 in *P. mume*. The reaction was performed in a 10-µL volume containing 5 µL of ‘KOD One PCR Master Mix’ (Toyobo Co., Ltd.), 0.3 µL of each primer (10 µM), 4.2 µL of ddH_2_O, and 0.2 µL of *P. mume* genomic DNA (10 ng). The PCR program was 98°C for 10 s, followed by 35 cycles of 95°C for 10 s, 61°C for 5 s, and 68°C for 15 s.

### 2.10. RNA-seq analysis

Total RNA was extracted from the dormant buds of ‘SC’ using a modified CTAB method, as previously described.^31,32^ The integrity and quality of the RNA were assessed by agarose gel electrophoresis. mRNA libraries were constructed from the RNAs extracted from ‘SC’ vegetative buds and sequenced on the DNBSEQ 2X150bp platform (Azenta, Tokyo, Japan), with 3 biological replicates. In addition, RNAseq data from ‘Nanko’ vegetative buds,^45^ collected on the same dates as those of ‘SC’, were included in this study.

Fastp was used to assess the quality of the mRNA sequencing data, perform adapter trimming, and filter for quality.^76^ Clean reads were then mapped to the *P. mume* ’Nanko’ haploid 1 genome using the STAR Aligner with default parameters.^88,89^ The number of reads mapped to each gene was calculated using the Subread package:featureCounts program.^90^ Raw read counts were normalized and converted to transcript per million (TPM) values. PCA was performed to profile gene expression patterns from different developmental stages between the vegetative buds of ‘Nanko’ and ‘SC’ during dormancy (Fig. S2). In addition, genes with low or no expression were filtered out using a threshold of 1 TPM.

Differentially expressed genes (DEGs) were identified by comparing the 2 parents, ‘Nanko’ and ‘SC’, at each stage during dormancy, as well as by comparing the stages within each cultivar to assessing cultivar and seasonal differences. DEGs were determined using the DESeq2 R package,^91^ following the filtering criteria of |log2FoldChange| >1 and a threshold of padj <0.05.

## 3. Results

### 3.1. Genome assembly of *P. mume* ‘Nanko’

The assembled diploid genome of ‘Nanko’ totals 478.7 Mb in scaffold length, with a scaffold N50 length of 28.1 Mb. This genome assembly achieves 99.1% complete BUSCO hits, indicating high quality (Table 1; Fig. S3). Haploid 1, distinguished by longer pseudo-chromosomes, has a total scaffold length of 245.8 Mb, including 8 pseudo-chromosomes and 306 scaffolds, in total 314 scaffolds. Its largest pseudo-chromosome is 47.1 Mb, with a scaffold N50 length of 28.1 Mb and a scaffold N90 count of 8, showing that over 90% of the HiFi reads were anchored in the 8 main pseudo-chromosomes (Table 1). In contrast, Haploid 2, featuring smaller pseudo-chromosomes, has a total scaffold length of 232.9 Mb, comprising 8 pseudo-chromosomes and 208 scaffolds, in total 216 scaffolds. Its largest pseudo-chromosome is 46.0 Mb, with a scaffold N50 length of 26.9 Mb and a scaffold N90 count of 8 (Table 1). The complete BUSCO scores for Haploid 1 and Haploid 2 are 99.0% and 98.2%, respectively (Table 1; Fig. S3). These metrics indicate that the diploid genome assembly is well-assembled at both the chromosome and haplotype-resolved levels, surpassing the quality and integrity of the *P. mume* V1.0 assembly (Table 1).^5^ The diploid genome assembly identifies 96,891 genes/alleles, with 43,400 and 41,933 genes located on the 8 pseudo-chromosomes of Haploid 1 and Haploid 2, respectively. In comparison, the *P. mume* V1.0 genome contains 22,227 genes on its main 8 pseudo-chromosomes,^5^ revealing an additional 21,173 and 19,706 well-assembled genes/alleles in the pseudo-chromosomes of *P. mume* ‘Nanko’ Haploid 1 and Haploid 2, respectively.

**Table 1.** Comparison of genome assembly statistics between *P. mume* ‘Nanko’ and *P. mume* V1.0 genomes (Zhang et al., 2012) and *P. mume* ‘Tortuosa’ (Zheng et al., 2022)

	Diploid	Haploid 1	Haploid 2	*P. mume* V1.0	*P. mume* ‘Tortuosa’
**Total length of scaffolds**	478.7Mb	245.8 Mb	232.9 Mb	237.1 Mb	237.7 Mb
**No. of scaffolds**	530	314	216	29,989	32
**Max. length of scaffolds**	47.1Mb	47.1 Mb	46.0 Mb	2.87 Mb	47.0 Mb
**Scaffold N50 length**	28.1 Mb	28.1 Mb	26.9 Mb	577.8 Kb	29.4 Mb
**Scaffold N50 count**	7	4	4	120	4
**Scaffold N90 length**	20.4 Mb	20.4 Mb	19.5 Mb	86.0 Kb	24.0 Mb
**Scaffold N90 count**	15	8	8	482	8
**GC content (%)**	38.27	38.33	38.20	37.00	37.59
**Complete Buscos (%)**	99.1	99.0	98.2	98.4	96.4

### 3.2. Comparative genomic analysis

**Fig. 1.**
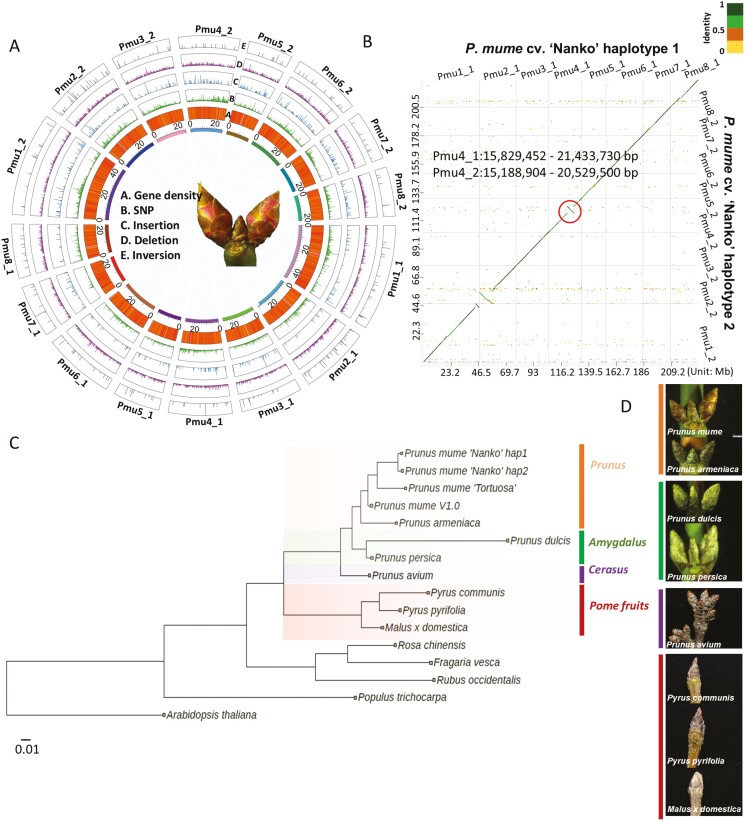
Genomic features of the *Prunus mume* ‘Nanko’ genome and the comparative genomic analysis of Prunus genome. (A) Genomic and variant features of *P. mume* cv. ‘Nanko’. The intermediate circles from the outer circle to the inner circle represent, gene densities (1), variant densities of single nucleotide polymorphism (2), insertions (3), deletions (4), and inversions (5) between 2 haploids. (B) Genomic synteny analysis between 2 haploids of *P. mume* ‘Nanko’. The colour scale ranges from yellow to green, indicating similarity from low to high. (C) Species tree analysis among horticultural crops of Rosaceae species and model plants, *Arabidopsis thaliana* and *Populus trichocarpa*. (D) Photograph of dormant bud in woody species of the Rosaceae family. Orange, green, purple, and brown indicate the subgenus *Prunus*, *Amygdalus*, *Cerasus*, and the pome fruit species in Rosaceae, respectively.

In *P. mume* ‘Nanko’, we have identified relatively long inversions occurring 2 haplotypes on the Pmu1, Pmu2, and Pmu4 chromosomes (Fig. 1A,B). A detailed examination focussed on Pmu4 confirmed the presence of a significant inversion. By aligning Pmu4_1 and Pmu4_2, the 2 pseudo-chromosomes, we observed a lengthy inversion between them spanning the interval of 15.829 Mb—21.433 Mb in Pmu4_1 and 15.188 Mb—20.529 Mb in Pmu4_2 (Fig. 1B). Further investigation involved aligning the raw assemblies of these pseudo-chromosomes to assess if subsequent assembly steps caused the inversion. The alignment revealed that the inversion persisted between Pmu4_1 and Pmu4_2 in the raw assemblies, suggesting it is not an artefact of the assembly process. PCR confirmation supported this observation, highlighting the uniqueness of the inversion to the ‘Nanko’ genome and its absence in ‘SC’ (Fig. S4A,B). Comparisons of the primary 8 pseudo-chromosomes of *P. mume* ‘Nanko’ Haploid 1 against the *P. mume* V1.0 genome^5^ and other *Prunus* species genomes (*P. armeniaca*, *P. persica*, *P. dulcis*, and *P. avium*)^57,58,60,92^ also detected the inversion in Pmu4 (Fig. S5). These findings underscore an exclusive inversion within the 15.8–21.4 Mb interval in Pmu4_1, distinguishing it from Pmu4_1 and other *Prunus* species (Fig. S5).

Species tree analysis, incorporating the ‘Nanko’ genome, delineated distinct clustering within the *Prunus* genus, particularly among the subgenera *Prunus*, *Amygdalus*, and *Cerasus* (Fig. 1C,D).^93,94^*P. mume* and *P. armeniaca* are positioned under the subgenus *Prunus*, while *P. dulcis* and *P. persica* categorized within the subgenus *Amygdalus*. *P. avium* is assigned to the subgenus *Cerasus*, and this clustering of species aligns with established studies on plant taxonomy and evolution (Fig. 1C).^93,94^ In addition, the outward appearance of dormant buds within each subgenus demonstrates a higher degree of resemblance (Fig. 1D). Within the clade of subgenus *Prunus*, the genomes of *P. mume* include *Prunus mume* V1.0, deriving from a wild accession in Tibet, China, believed to be the progenitor of *P. mume*.^5,30^ Following this is the ornamental weeping cultivar ‘Tortuosa’ from China,^67^ and the most recent development is the main fruit-producing cultivar ‘Nanko’ in Japan (Fig. 1C).

### 3.3. Segregation and correlation of vegetative bud dormancy-related phenotypes in NKSC F_1_ population

**Fig. 2.**
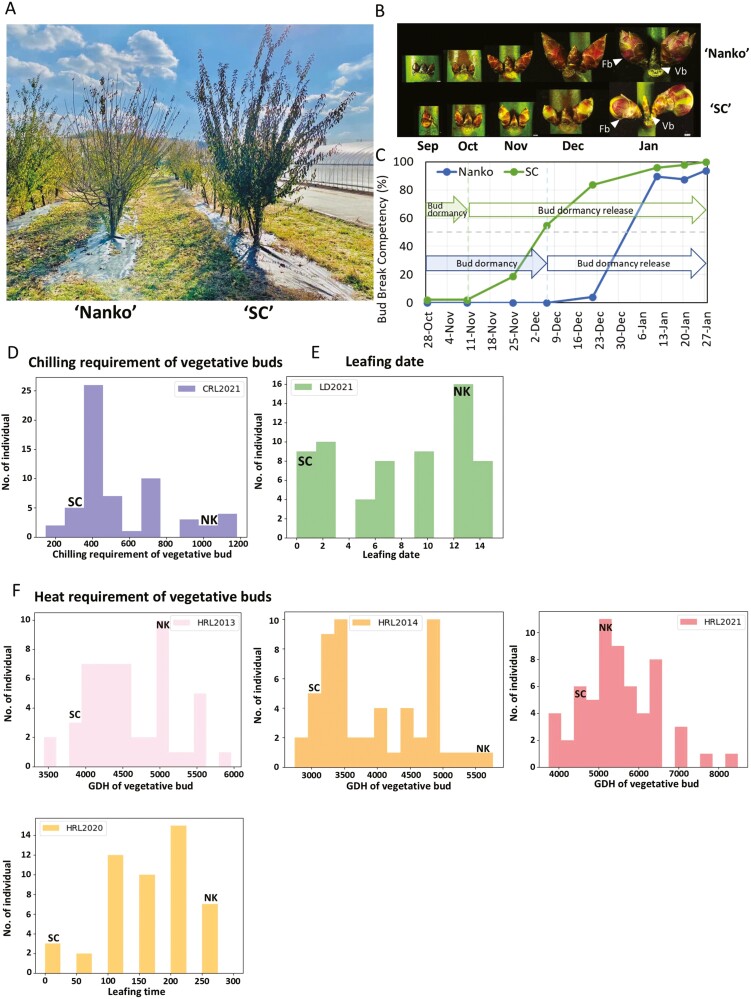
Two Japanese apricot cultivars with contrasting CR, high-chill ‘Nanko’ and low-chill ‘SC’, and the frequency distribution of bud dormancy-related traits in the NKSC F1 population. (A) Photograph of ‘Nanko’ and ‘SC’ trees taken on December 23^rd^, 2020. (B) Development stages of ‘Nanko’ and ‘SC’ vegetative (leaf) and flower buds during dormancy. (C) Seasonal variation in bud break competency rate (%) of vegetative buds of ‘Nanko’ and ‘SC’. (D) Frequency distribution of chilling requirement of vegetative buds (E) Frequency distribution of leafing date, and (F) Frequency distribution of heat requirement of vegetative buds, evaluated by growing degree hours (GDH) and leafing time (LT), respectively. All phenotypes were observed during 2020–2021 and 2021–2022 seasons, with HRL2013 and HRL2014 calculated using the raw data from a previous study (Kitamura et al., 2019). NK stands for ‘Nanko’. ‘Fb’ refers to flower buds, and ‘Vb’ refers to vegetative buds.

The phenotypic data of vegetative bud dormancy-related traits for the ‘NKSC’ F_1_ population is depicted in Fig. 2. In the 2021-22 season, CR of vegetative buds (CRL2021) were 387 CH for ‘SC’ and 1044 CH for ‘Nanko’. Within the F_1_ segregating population, CRL2021 ranged from 145 to 1183 CH (Fig. 2D). LD (LD2021) for ‘SC’ and ‘Nanko’ were 0 and 12 days after the first leafing, respectively, while the F_1_ segregating population showed LD variation from 0 to 15 days post-initial leafing (Fig. 2E). For HR phenotyping, 2 methods were employed: GDH and LT (days to leafing in forcing condition) (see Materials and Methods). For the 2013-14 season, GDH-based HR (HRL2013) were 3,926 and 5,132 for ‘SC’ and ‘Nanko’, respectively, with the F_1_ segregating population ranging from 3,439 to 5,967 GDH (Fig. 2F). In the 2014–2015 season, HRL2014 were 3,138 and 5,768 GDH for ‘SC’ and ‘Nanko’, respectively, with the F_1_ segregating population ranging from 2,738 to 5,768 GDH (Fig. 2F). In the 2021–2022 season, GDH-based HR (HRL2021) were 4,628 and 5,238 GDH for ‘SC’ and ‘Nanko’, respectively (Fig. 2F). Using the LT method for HR phenotyping in the 2020-21 season, HRL2020 for ‘SC’ and ‘Nanko’ were 24 and 262 h, respectively, with the F_1_ segregating population ranging from 24 to 262 h (Fig. 2F).

Spearman’s rank correlations among observed phenotypes across seasons are presented in Table 2. Correlation coefficient between CRL and LD ranged from 0.27 to 0.83. Particularly, CRL2013 exhibited notable correlations with LD2013, LD2014, and LD2021, with coefficients of 0.83, 0.78, and 0.51, respectively (Table 2). However, significant positive correlations (*r* ≥ 0.6) between CRL and HRL were only observed for CRL2013 with HRL2014 and HRL2020, with coefficients of 0.61 and 0.69, respectively (Table 2). Moreover, LD showed significant positive correlations with HRL, especially LD2013 and LD2014, which exhibited notable associations with HRL2014 and HRL2020. Remarkably, LD2014 demonstrated an exceptionally high correlation (*r* = 0.92) with HRL2014 (Table 2).

**Table 2.** Spearman’s rank correlations for vegetative bud dormancy-related phenotype scores in the *P. mume* ‘NKSC’ F_1_ population. Correlation coefficient values in shaded by orange background indicate significance at *P* < 0.01.

	CRL2013	CRL2014	CRL2021	LD2013	LD2014	LD2021	HRL2013	HRL2014	HRL2020	HRL2021
**CRL2013**	1	0.71	0.61	0.83	0.78	0.51	0.16	0.61	0.69	-0.06
**CRL2014**	-	1	0.56	0.67	0.53	0.42	0.31	0.23	0.47	-0.03
**CRL2021**	-	-	1	0.46	0.46	0.27	0.05	0.27	0.46	-0.45
**LD2013**	-	-	-	1	0.77	0.42	0.59	0.62	0.66	-0.03
**LD2014**	-	-	-	-	1	0.43	0.34	0.92	0.78	-0.08
**LD2021**	-	-	-	-	-	1	0.11	0.34	0.42	0.64
**HRL2013**	-	-	-	-	-	-	1	0.26	0.28	0.08
**HRL2014**	-	-	-	-	-	-	-	1	0.69	-0.04
**HRL2020**	-	-	-	-	-	-	-	-	1	-0.02
**HRL2021**	-	-	-	-	-	-	-	-	-	1

### 3.4. Identification of vegetative bud dormancy -associated QTLs using *P. mume* ‘Nanko’ genome

**Fig. 3.**
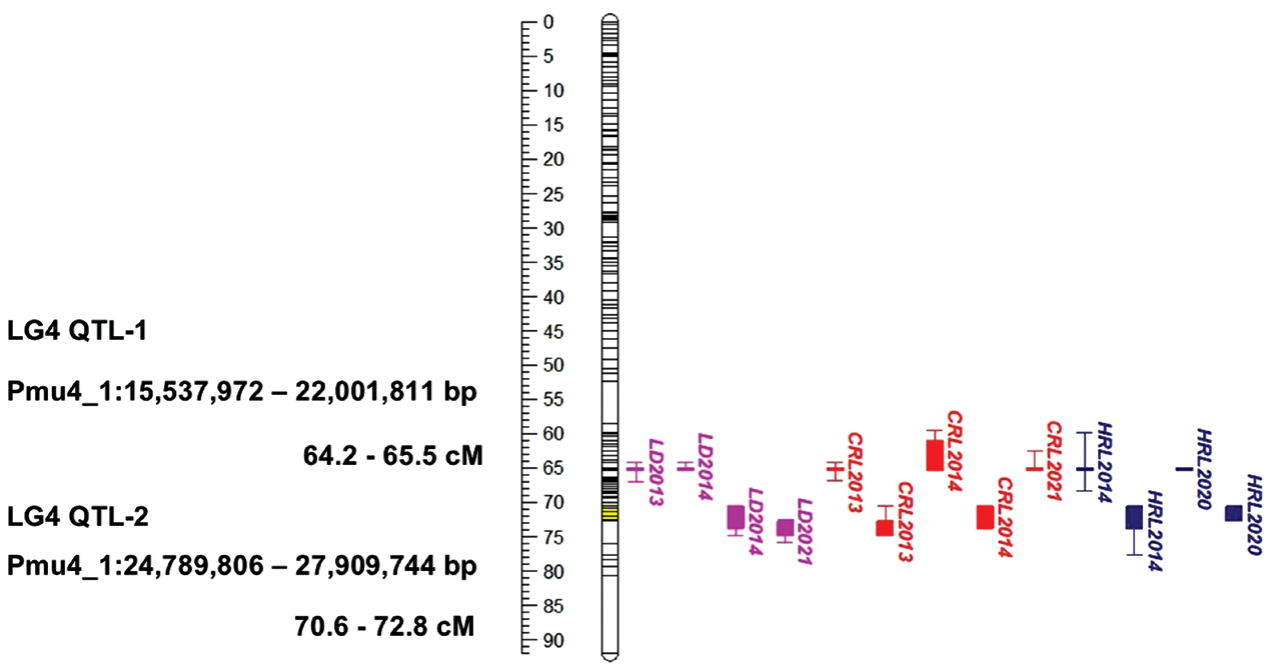
Location of QTLs detected in the genetic linkage map 4 (LG4) of NKSC. Pink, red, and navy-blue indicate QTLs controlling leafing date (LD), chilling requirement of leaf buds (CRL), heat requirement for bud break of vegetative buds (HRL), respectively. Numbers represent the observed season year. Boxes and bars indicate 1- and 1.5-LOD confidence intervals from peak LOD values.

Using the *P. mume* ‘Nanko’ haploid 1 genome as a reference, we constructed a maternal genetic map (‘Nanko’) consisting of 1,172 SNPs and spanning 583 cM, and a paternal genetic map (‘SC’) comprising 1,422 SNPs and spanning 621 cM. The average interval distance between SNP markers was 0.50 cM for the maternal map and 0.44 cM for the paternal map. QTLs associated with vegetative bud dormancy traits were identified in LG4 and LG7, consistent with previous findings.^13^ In the ‘Nanko’ LG4 map, 2 distinct QTL regions (QTL-1 and QTL-2) were identified, but these were not observed in the ‘SC’ LG4 map. This finding aligns with our earlier study^13^ (Fig. 3 and Table 3)

**Table 3.** Identified QTLs for bud dormancy-related traits on LG4 of ‘Nanko’

Trait	Year	LG	Position (cM)	Peak LOD	Nearest Marker	PVE (%)
**Leafing date (LD)**	2013	4	65.15	13.18	Pmu4_1:15,537,972	45.2
	2014	4	65.15	9.89	Pmu4_1:15,537,972	36.9
	2014	4	70.62	9.44	Pmu4_1:27,801,352	35.3
	2021	4	72.79	3.82	Pmu4_1:24,789,806	25.4
**Chilling requirement of vegetative bud (CRL)**	2013	4	65.15	13.60	Pmu4_1:15,537,972	46.2
	2013	4	72.79	10.89	Pmu4_1:24,789,806	39.1
	2014	4	65.15	4.47	Pmu4_1:15,537,972	18.6
	2014	4	72.79	3.91	Pmu4_1:24,789,806	16.5
	2021	4	65.15	3.85	Pmu4_1:15,537,972	26.7
**Heat requirement of vegetative bud (HRL)**	2014^1^	4	65.15	7.30	Pmu4_1:15,537,972	28.8
	2014^1^	4	70.62	7.25	Pmu4_1:27,801,352	28.6
	2020^2^	4	65.15	3.75	Pmu4_1:15,537,972	27.4
	2020^2^	4	72.58	3.75	Pmu4_1:27,909,744	27.4

^1^The heat requirement of vegetative bud was evaluated by growth degree hour (GDH).

^2^The heat requirement of vegetative bud was determined by measuring the total number of day for bud break rate to exceed 40% after cultivation at 23°C and a photoperiod of 16/8 h.

LG4 QTL-1 was identified for LD2013, and LD2014, with the highest LOD score recorded for LD2013 (LOD score: 13.18 and phenotypic variance explained (PVE): 45.2%) (Table 3). The LG4 QTL-2 was detected for LD2014 and LD2021, with the highest peak LOD value for LD2014 (LOD score: 9.44 and PVE: 35.3%) (Table 3). In addition, LG4 QTL-1 was detected for CRL2013, CRL2014, and CRL2021, with the highest LOD score for CRL2013 (LOD score: 13.60 and PVE: 46.2%) (Table 3). For CRL2013 and CRL2014, the LG4 QTL-2 was also identified, with CRL2013 showing higher LOD and PVE scores of 10.89 and 39.1%, respectively (Table 3). Among HRL2013, HRL2014, HRL2020, and HRL2021, LG4 QTL-1 and QTL-2 were only detected for HRL2014 and HRL2020 (Fig. 3 and Table 3). Notably, HRL2014 exhibited higher peak LOD scores for both LG4 QTL-1 (LOD score: 7.30) and LG4 QTL-2 (LOD score: 7.25) compared to HRL2020 (Table 3). The overlapping interval for LG4 QTL-1 across all LD, CRL, and HRL traits spans from 64.2 to 65.5 cM, corresponding to the physical position Pmu4_1:15,537,972 – 22,001,811 bp (Fig. 3). The LG4 QTL-2 interval ranges from 70.6 to 72.8 cM, corresponding to the physical position of Pmu4_1:24,789,806 – 27,909,744 bp (Fig. 3).

In our previous study, a QTL for CRL was detected on LG7 in the ‘SC’ map but not in the ‘Nanko’ map.^13^ LG7 QTL for CRL2013 had LOD and PVE scores of 4.33 and 17.9%, respectively. In addition, for HRL2020, an LG7 QTL was identified within a similar interval as the LG7 QTL for CRL2013, with peak LOD and PVE scores of 6.12 and 40.6%, respectively (Table 4). For HRL2020, the LG7 QTL exhibited a higher LOD score compared to the LG4 QTL (Table 3 and Table 4). The overlapping LG7 QTL intervals for CRL and HRL span from 37.4 to 40.3cM, corresponding to the physical map position Pmu7_1:16,236,530 – 17,709,773 bp (Table 4).

**Table 4.** Identified QTLs for bud dormancy-related traits on LG7 of ‘SC’

Trait	Year	LG	Position (cM)	Peak LOD	Nearest Marker	PVE (%)
**Chilling requirement of vegetative bud (CRL)**	2013	7	40.27	4.33	Pmu7_1:17,709,773	17.9
**Heat requirement of vegetative bud (HRL)**	2020^a^	7	38.99	6.12	Pmu7_1:16,838,992	40.6

^a^The heat requirement of vegetative bud was determined by measuring the total number of day for bud break rate to exceed 40% after cultivation at 23°C and a photoperiod of 16/8 h.

### 3.5. Haplotype analysis can refine the genomic regions linked to vegetative bud dormancy traits

**Fig. 4.**
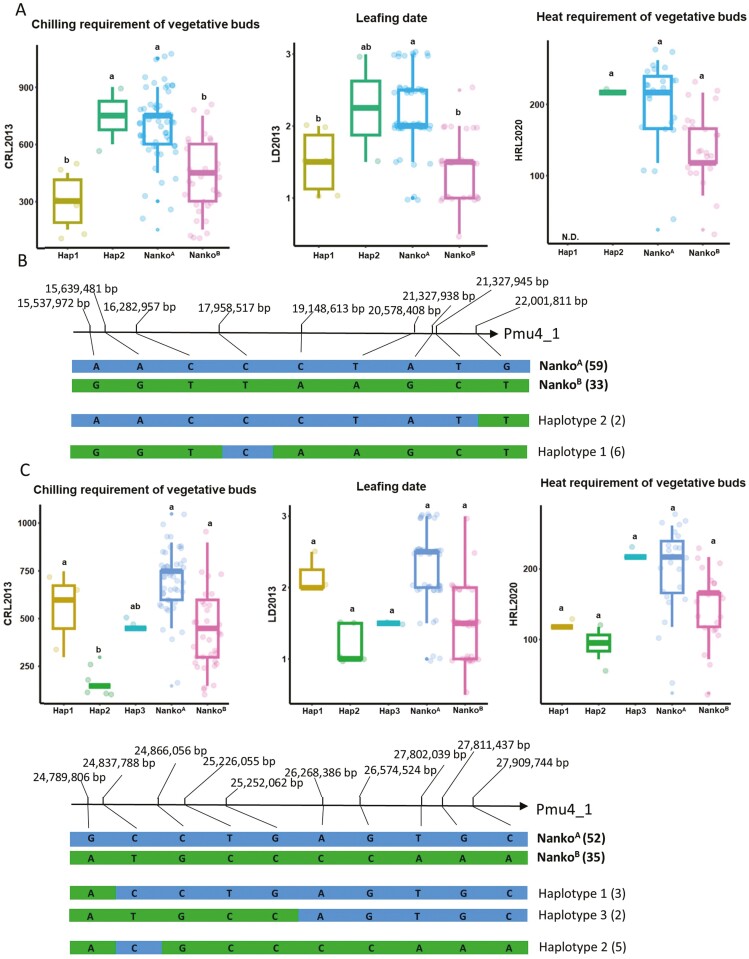
Haplotype analysis of the LG4 QTL-1 and -2 in ‘Nanko’ using CRL2013, LD2013 and HRL2020 phenotype data. (A) Box plots depicting phenotypic values for various haplotype groups within the LG4 QTL-1 interval. (B) Haplotype block constructed using SNP markers in the LG4 QTL-1 region. (C) Box plots illustrating phenotypic values for different haplotype groups within the LG4 QTL-2 interval. (D) Haplotype block derived from SNP markers in the LG4 QTL-2 region. Haplotypes with fewer than 2 individuals were excluded from this analysis. N.D. means no corresponding phenotype data.

We first examined the correlation between distinct recombined haplotypes from ‘Nanko’ and vegetative bud dormancy-related traits within the LG4 QTL-1 and -2 intervals in NKSC F_1_ population. In the LG4 QTL-1 interval, individuals with haplotype 2 showed significantly higher CR, delayed LD, and increased HR compared to those with haplotype 1 based on CRL2013, LD2013, and HRL2020 (Fig. 4A,B). Similar associations were observed for traits from other seasons (Fig. S6). The regions with most strongly linked to these traits are Pmu4_1:15,537,972-16,282,957 bp and Pmu4_1:19,148,613-21,327,945 bp.

In the LG4 QTL-2 region, individuals with haplotypes 1, and 3 had higher CR, HR and delayed LD compared to those with haplotype 2 (Fig. 4C,D, Fig. S7). The region most significantly associated with these traits is Pmu4_1:26,268,386 -27,909,744 bp. Within these 3 high-confidence LG4 QTL intervals, we identified a total of 894 genes (Table S5). The identified 5.6 Mb chromosomal inversion (Pmu4_1:15,829,452-21,433,730 bp) fully overlapped with the LG4 QTL-1 region, suggesting a linkage relationship. PCR analysis confirmed that individuals with the inversion variant genotype (I, indicating the inversion allele from ‘Nanko’) exhibited lower CR, HR and LD, while those with the non-inversion allele from ‘Nanko’ (i^N^) showed significantly higher CR, HR and delayed LD in several seasons (Fig. S8).

For LG7 QTL, we examined the correlation between distinct recombined haplotypes from ‘SC’ and dormancy traits. Individuals with haplotypes 2 and 3 had higher CRL2013 and HRL2020 compared to those with haplotype 1 (Fig. S9). The regions most strongly associated with these traits were identified at Pmu7_1:16,236,530-16,479,089 bp, and Pmu7_1:16,602,754-17,442,592 bp (Fig. S9). In this region of ‘Nanko’ haploid 1, we identified 197 genes, listed in Table S6.

### 3.6. Identification of candidate genes in LG4 and LG7 refined QTL intervals

To further refine the list of candidate genes, we filtered genes within the refined intervals using the following criteria:

1. Genes expressed in vegetative buds (≥1 Transcripts Per Million, TPM).
2. Genes showing differential expression between ‘Nanko’ and ‘SC’ and/or in seasonal changes.
3. Genes with variants in coding sequence (CDS) regions correlated with bud dormancy traits.

Genes meeting both criteria (1) and (2) or both criteria (1) and (3) were defined as candidate genes within the refined QTL regions. Among the 573 genes within the refined LG4 QTL-1 intervals, 203 genes were expressed in vegetative buds during dormancy (Table S5). Of these, 146 genes showed differential expression in monthly comparison between ‘Nanko’ and ‘SC’ and/or in seasonal changes (Table S5). In the LG4 QTL-2 refined intervals, 73 out of 321 genes were expressed in vegetative buds during dormancy (Table S5), with 43 of these genes showing differential expression (Table S5). In addition, 1,659 SNPs, 23 small indels, and 19 deletions showing high correlations with bud dormancy traits are located within CDS regions. For the 197 genes within the high-confidence LG7 QTL intervals, 182 genes were expressed, and 129 of these exhibited differential expression (Table S6). In the refined LG7 region, there are 152 SNPs, 6 small indels, and 1 deletion highly correlated with dormancy traits located within CDS regions.

Thus, the number of candidate genes in the refined LG4 QTL-1 and QTL-2 intervals meeting the criteria were 157 and 38, respectively. Annotated genes among these (127 for LG4 QTL-1 and 31 for LG4 QTL-2) are listed in Table S5. Based on their annotation and expression patterns, we propose the following genes as examples of candidate genes:

1. *MAINTENANCE OF MERISTEMS* (*PmuMAINs*, Pmu.4_1G418880, Pmu.4_1G418890, and Pmu.4_1G418891)
2. *PROTEIN TRANSLATION FACTOR SUI1* (*PmuSUI1*, Pmu.4_1G419380)
3. *AUTOPHAGY-RELATED PROTEIN 8C-LIKE* (*PmuATG8CL*, Pmu.4_1G430470)
4. *NAC domain-containing protein 2* (*PmuNAC2*, Pmu.4_1G439330)
5. *DELAY OF GERMINATION 1-like 3* (*PmuDOG1-like 3*, Pmu.4_1G439570).

**Fig. 5.**
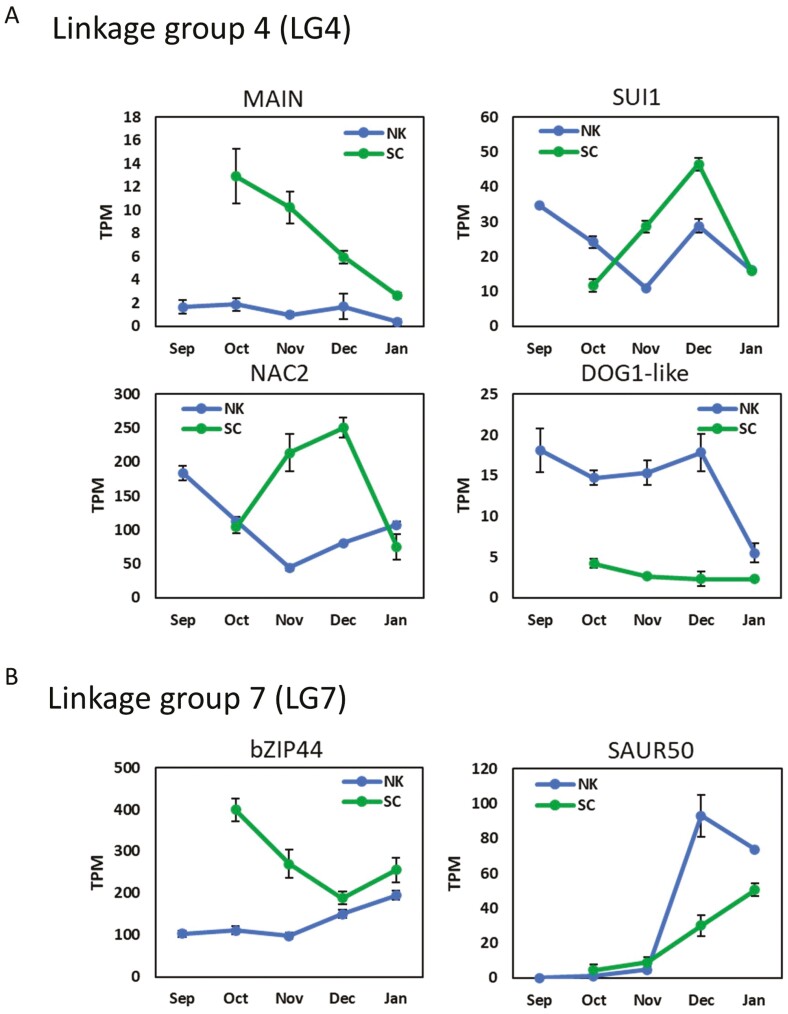
Expression profiles of candidate genes within the refined intervals of the vegetative bud dormancy-associated QTLs on LG4 and LG7. (A) The expression levels of MAINTENANCE OF MERISTEMS (PmuMAIN, Pmu.4_1G418880), Protein translation factor SUI1 (PmuSUI1, Pmu.4_1G419380), NAC domain-containing protein 2 (PmuNAC2, Pmu.4_1G439330), and Protein DOG1-like 3 (PmuDOG1-like 3, Pmu.4_1G439570) within the LG4 QTL interval. (B) Expression levels of bZIP transcription factor 44 (PmubZIP44, Pmu.7_1G709280) and SMALL AUXIN UP-REGULATED RNA 50 (PmuSAUR50, Pmu.7_1G709930) within the LG7 QTL interval. Expression values are presented as TPM (*n* = 3). NK denotes ‘Nanko’.

The expression patterns of these genes are shown in Fig. 5A. *MAIN* exhibited higher expression in ‘SC’ vegetative buds compared to ‘Nanko’ and decreased towards the bud break period. *PmuSUI1* (Pmu.4_1G419380), located at the inversion breakpoint, increased expression from November to December, with earlier up-regulation in ‘SC’ compared to ‘Nanko’ (Fig. 5A). A small deletion (AGG/AG) in the CDS of *MAIN* (Pmu.4_1G418890) correlated with higher CR and HR and delayed LD (Supplementary Fig. S10A). An amino acid change (Pro52Leu) in *PmuATG8CL* (Pmu.4_1G430470) was associated with decreased CR (Fig. S10B). *PmuNAC2* (Pmu.4_1G439330) had higher expression in ‘SC’ and increased upon dormancy release (Fig. 5A). Its CDS contains a SNP variant associated with an amino acid change (Gln4Arg), and individuals carrying these variants exhibited lower CR and HR and earlier LD (Fig. S10C). *PmuDOG1-like 3* (Pmu.4_1G439570) showed significantly lower expression in ‘SC’ compared to ‘Nanko’ during dormancy progression (Fig. 5A). In ‘Nanko’, the expression of *PmuDOG1-like 3* sharply decreased when CR was fulfilled (December) (Fig. 5A).

In the refined LG7 QTL, we identified 159 candidate genes, with 116 annotated (Table S6). Expression patterns of selected genes such as *bZIP TRANSCRIPTION FACTOR 44* (*PmubZIP44*, Pmu.7_1G709280) and *SMALL AUXIN UP-REGULATED RNA 50* (*PmuSAUR50*, Pmu.7_1G709930), are shown in Fig. 5B. *PmubZIP44* exhibited high expression levels in ‘SC’ before dormancy and after dormancy release (in October and January), while ‘Nanko’ showed lower expression during dormancy (Fig. 5B). *PmuSAUR50* (Pmu.7_1G709930) showed a dramatic increase in expression in both ‘SC’ and ‘Nanko’ during December and January (Fig. 5B; Table S6).

## Discussion

### 4.1. ‘Nanko’ genome assembly provides insights into *P. mume* evolution

Generally, *P. mume* shows the closest genetic relationship with *P. armeniaca*, rather than with other species of the subgenus (i.e. *Amygdalus* and *Cerasus*),^28,93,94^ which is consistent with the subgenus-specific morphological similarities in bud structure (Fig. 1C,D). Our constructed *P. mume* ‘Nanko’ genome also supports previous research, which have demonstrated that *P mume* shares greater genome similarity with plum and apricot.^95,96^ In addition, our results suggest that the divergence between the Japanese and Chinese groups of *P. mume* occurred in an evolutionary sequence (Fig. 1C). *P. mume* V1.0 is from ancient wild germplasm in Tibet,^5^ while *P. mume* ‘Tortuosa’ belongs to the weeping ornamental cultivar from the Chinese group^67^ (Fig. 1C). This finding aligns with previous genomic studies, supporting the hypothesis that the origin of Japanese apricot traces back to Tibet, spreading eastward through the Yangtze River basin system and the Pearl River basin systems to reach Japan.^5,29^ The results of this study further reinforce the hypothesis that the evolution of *P. mume* in Japanese germplasm occurred later and exhibited a unique genetic background due to geographical isolation.

### 4.2. Chromosome-level inversion in LG4 is linked to bud dormancy QTL in *P. mume* ‘Nanko’

In genomic studies of *Prunus* species, numerous reports have highlighted the common occurrence of chromosomal inversions among different species and cultivars.^97–99^ For example, a significant inversion spanning 7,588 kb on chromosome 5 has been noted, potentially contributing to distinctive traits in *P. tenella*, such as dwarfing and freezing resistance.^99^ In addition, a structural variation-based genome-wide association study (SV-based GWAS) identified a substantial 1.67-Mb heterozygous inversion that perfectly co-segregates with the flat-fruit shape in peach.^98^ In this study, we identified a 5.6 Mb heterozygous inversion in one of *P. mume* ‘Nanko’ haploids on LG4, a feature exclusive to ‘Nanko’. Interestingly, this chromosomal inversion overlaps with the LG4 QTL (Figs 1B and 3). In NKSC F_1_ population, individuals carrying the inversion variant exhibit lower CR and HR, as well as earlier LD, compared to those with the non-inversion allele. One possible explanation for these results is that even though ‘Nanko’ is high-chill cultivar, genotypes of the loci is heterozygous for higher and lower-chill allele and the performance of the inversion allele (I) may contribute to lower-chill while non-inversion allele (i^N^) may lead to higher-chill allele. On the contrary, SC appeared to have i^N^ allele but low chill trait of SC may be primarily controlled by the locus other than the LG4 QTL locus. Indeed, the LG4 QTL was not detected in ‘SC’ genetic map. Further investigation is necessary to determine if the identified inversion is indeed responsible for the LG4 QTL.

### 4.3. The potential candidate genes in the refined LG4 and LG7 intervals

Among the candidate genes identified based on our criteria, several stand out as highly probable. Within the refined LG4 intervals, we identified the *MAIN* gene, which shows expression changes related to dormancy. In Arabidopsis, mutations in *MAIN* cause disordered shoot apical meristem (SAM), underscoring its vital role in cell division and differentiation.^100,101^ Notably, the *PmuSUI1* (Pmu.4_1G419380) gene is located precisely at the breakpoint of the chromosomal inversion (Table S5). In rice, a *SUI1* homolog encoding a translation initiation factor known as eIF-1, regulates ion homeostasis and inhibits intracellular accumulation of reactive oxygen species by up-regulating oxido-reductase. Transgenic rice overexpressing eIF-1 shows improved growth conditions under stress compared to the wild type.^102^ Further investigation into *PmuSUI1* could shed light on the relationship between the inversion event and bud dormancy trait. Another noteworthy candidate gene is *PmuATG8CL*. In Arabidopsis, ATG proteins are involved in lipid droplet degradation, contributing to seed germination.^103^ The rice mutant *Osatg7*, which lacks autophagy, exhibits male sterility and reduced triacylglycerols (TAGs) in anthers.^104^ Similarly, Arabidopsis mutants *atg2* and *atg5* show decreased leaf TAGs.^105^ Recent studies suggest that lipid body accumulation is a key metabolic change related to bud dormancy.^45^

Within the LG4 QTL-2 interval, we propose 2 candidate genes, *PmuNAC2* and *PmuDOG1*. In cowpea (*Vigna unguiculata* (L.) Walp.), overexpression of *VuNAC2* promotes growth and reproductive development, imparting resistance to various abiotic stresses, which results at increased leaf area, thicker stems, early flowering, higher pod yield, and seed weight.^106^ These findings imply that the differential expression and/or amino acid changes in PmuNAC2 (Pmu.4_1G439330) may influence activity, growth, and development in vegetative buds in *P. mume*. Arabidopsis DOG1 is a known regulator of primary dormancy (PD) and works with ABA to delay seed germination.^107,108^ Functional studies and GWAS in *Arabidopsis* have highlight DOG1’s crucial role in regulating seed germination, response to cold, and flowering time control across different germplasms.^109–111^ Therefore, we suppose *PmuDOG1-like 3* in *P. mume* may act as a regulator of temperature response (e.g. chilling requirement) in buds, akin to its role in regulating seed dormancy, thermal response, and flowering in Arabidopsis.

From the refined LG7 QTL interval, we highlight 2 genes as candidates controlling bud dormancy traits: *PmubZIP44* and *PmuSAUR71*. Previous reports indicated significant differences in ABA levels between high and low CR individuals of NKSC F_1_ population,^45^ suggesting a close relationship between ABA metabolism and QTL in this population. In soybean, overexpression of *GmbZIP44* reduces ABA sensitivity, acting as a negative regulator of ABA signalling.^112^ Hence, *PmubZIP44* might control vegetative bud dormancy by inhibiting ABA signalling. In sweet cherry (*P. avium* L.), *SAUR71* (*PavSAUR71*, PAV04_REGINAg0203401) was identified as a candidate gene in flowering-associated QTL in LG4, showing differential expression between cultivars with contrasting CR during flower bud dormancy release.^20^*SAUR* genes, the largest early auxin-responsive gene family, play crucial roles in regulating plant growth and development.^27^ For example, in Arabidopsis, members of the SAUR41 subfamily, including *SAUR40*, *SAUR41*, *SAUR71*, and *SAUR72*, regulate cell expansion/elongation in cotyledons and hypocotyls.^27,59,113–115^ In addition, SAUR50 suppresses PP2C-D1 activity and promotes cell expansion in Arabidopsis.^113–115^ In *P. mume*, *PmuSAUR50* (Pmu.7_1G709930) was up-regulated from dormancy to dormancy release, suggesting its rapid up-regulation might inhibit ABA signal transduction, resulting in varied ABA sensitivity and regulation of bud dormancy.

In conclusion, we have constructed a haploid-phased, high-quality *P. mume* genome using the Japanese cultivar, Nanko, enabling us to decipher the identified bud dormancy-related QTLs. This study provides a fundamental resource for future gene (allele) discovery and characterization in *P. mume*, facilitating genomic and genetic studies on bud dormancy regulation in fruit tree species of *Prunus*.

## Supplementary Material

dsae034_suppl_Supplementary_Tables_S1-6

dsae034_suppl_Supplementary_Figures_S1-S10

## Data Availability

All pertinent information is included within the manuscript and its accompanying materials. The RNA-seq data concerning the ‘SC’ vegetative buds produced in this study were archived in the Gene Expression Omnibus database of the National Center for Biotechnology Information (http://www.ncbi.nlm.nih.gov/geo) under the accession number PRJNA1074819. The *Prunus mume* ‘Nanko’ genome assembly was archived in the Genome Database for Rosaceae (GDR) (https://www.rosaceae.org/) under the accession number tfGDR1076.
