# Supplementary material for: The haplotype-phased genome assembly facilitated the deciphering of the bud dormancy-related QTLs in Prunus mume
Source: DNA Res. 2024 Dec 4;32(1):dsae034. doi: 10.1093/dnares/dsae034 (PMC11747360; doi:10.1093/dnares/dsae034)
Supplement: dsae034_suppl_Supplementary_Figures_S1-S10 [file dsae034_suppl_supplementary_figures_s1-s10.pptx]

## Slide 1
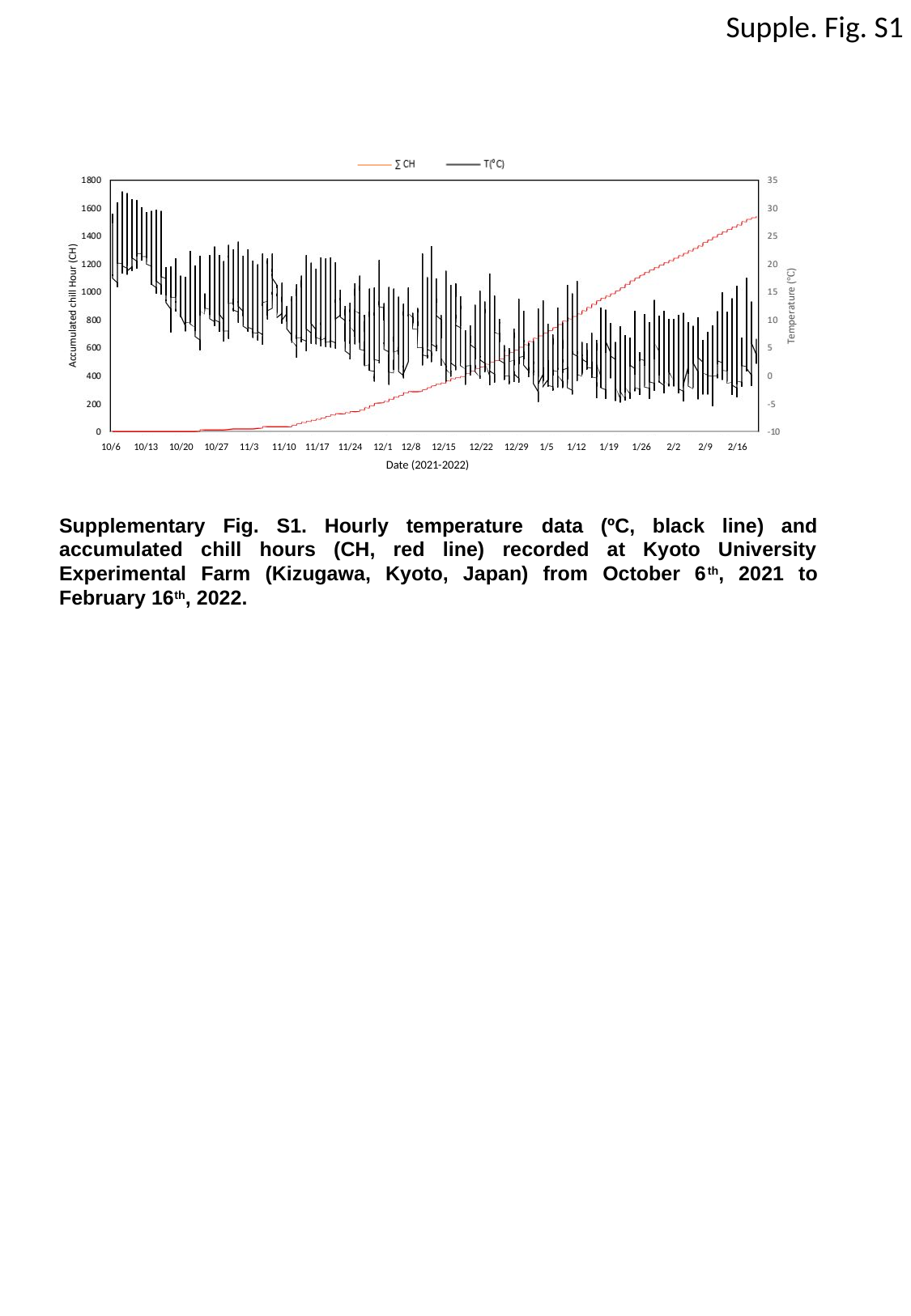

Supple. Fig. S1
10/6 10/13 10/20 10/27 11/3 11/10 11/17 11/24 12/1 12/8 12/15 12/22 12/29 1/5 1/12 1/19 1/26 2/2 2/9 2/16
Date (2021-2022)
Supplementary Fig. S1. Hourly temperature data (ºC, black line) and accumulated chill hours (CH, red line) recorded at Kyoto University Experimental Farm (Kizugawa, Kyoto, Japan) from October 6th, 2021 to February 16th, 2022.

## Slide 2
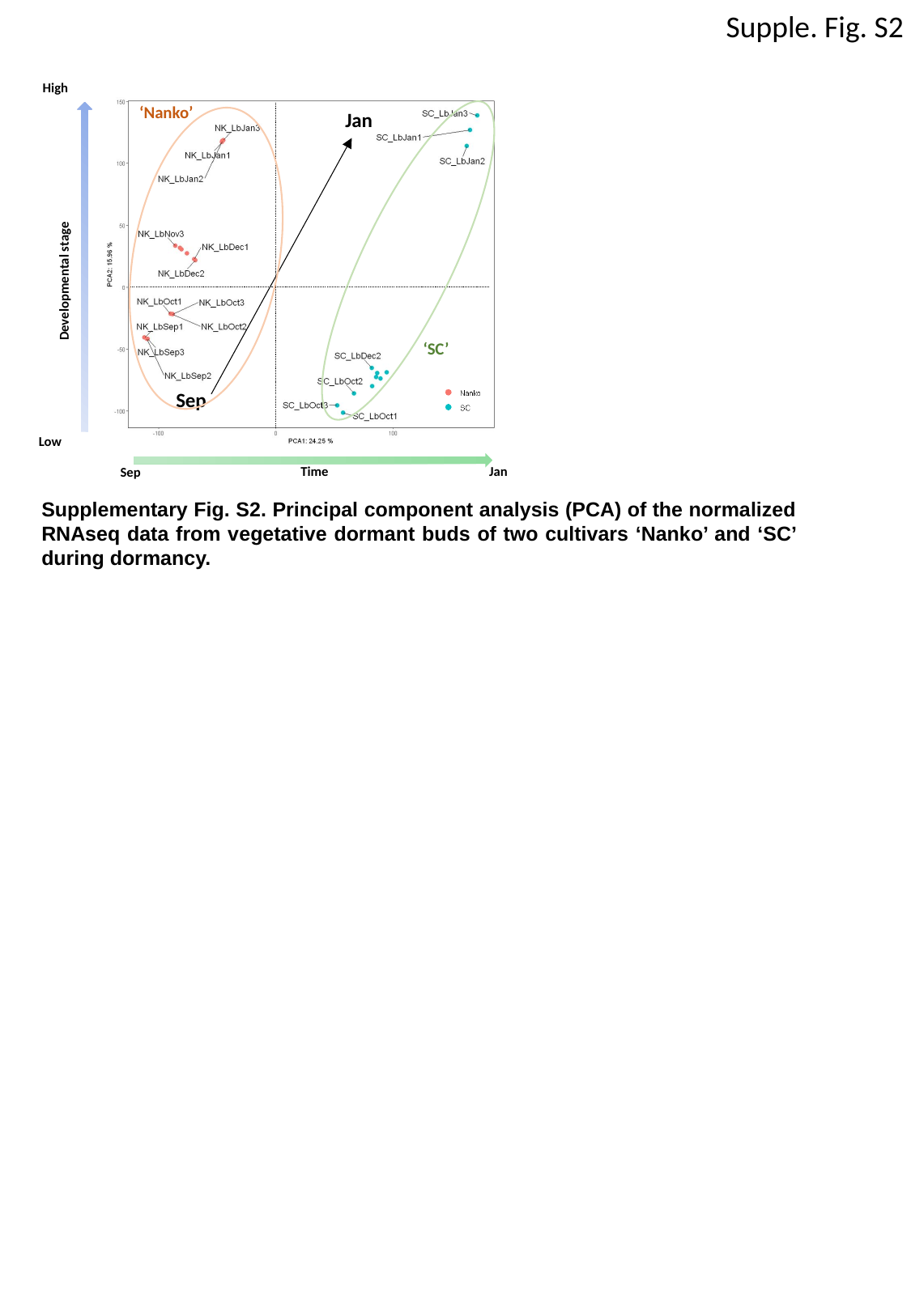

Supple. Fig. S2
High
Jan
Developmental stage
Sep
Low
Time
Jan
Sep
‘Nanko’
‘SC’
Supplementary Fig. S2. Principal component analysis (PCA) of the normalized RNAseq data from vegetative dormant buds of two cultivars ‘Nanko’ and ‘SC’ during dormancy.

## Slide 3
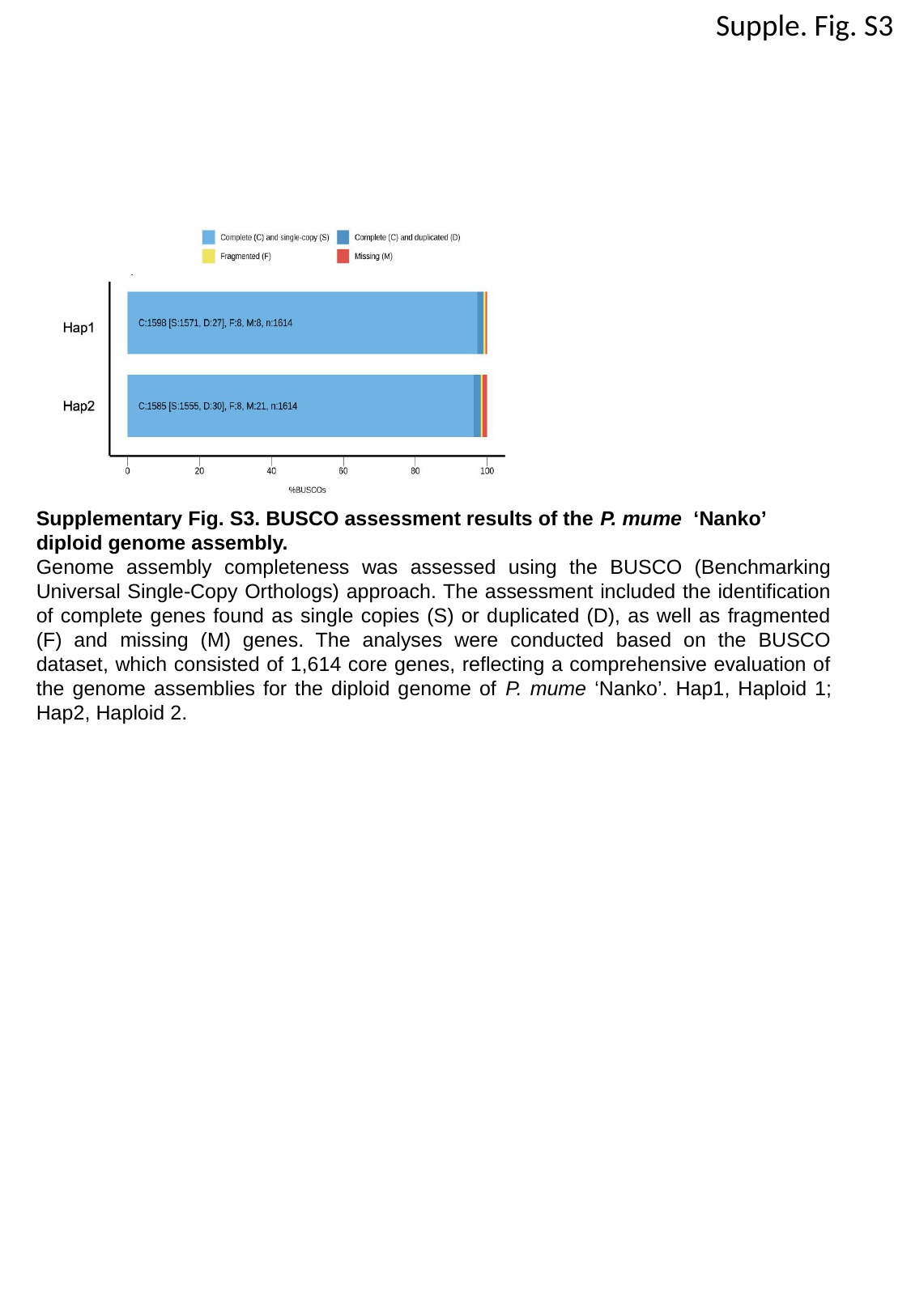

Supple. Fig. S3
Supplementary Fig. S3. BUSCO assessment results of the P. mume ‘Nanko’ diploid genome assembly.
Genome assembly completeness was assessed using the BUSCO (Benchmarking Universal Single-Copy Orthologs) approach. The assessment included the identification of complete genes found as single copies (S) or duplicated (D), as well as fragmented (F) and missing (M) genes. The analyses were conducted based on the BUSCO dataset, which consisted of 1,614 core genes, reflecting a comprehensive evaluation of the genome assemblies for the diploid genome of P. mume ‘Nanko’. Hap1, Haploid 1; Hap2, Haploid 2.

## Slide 4
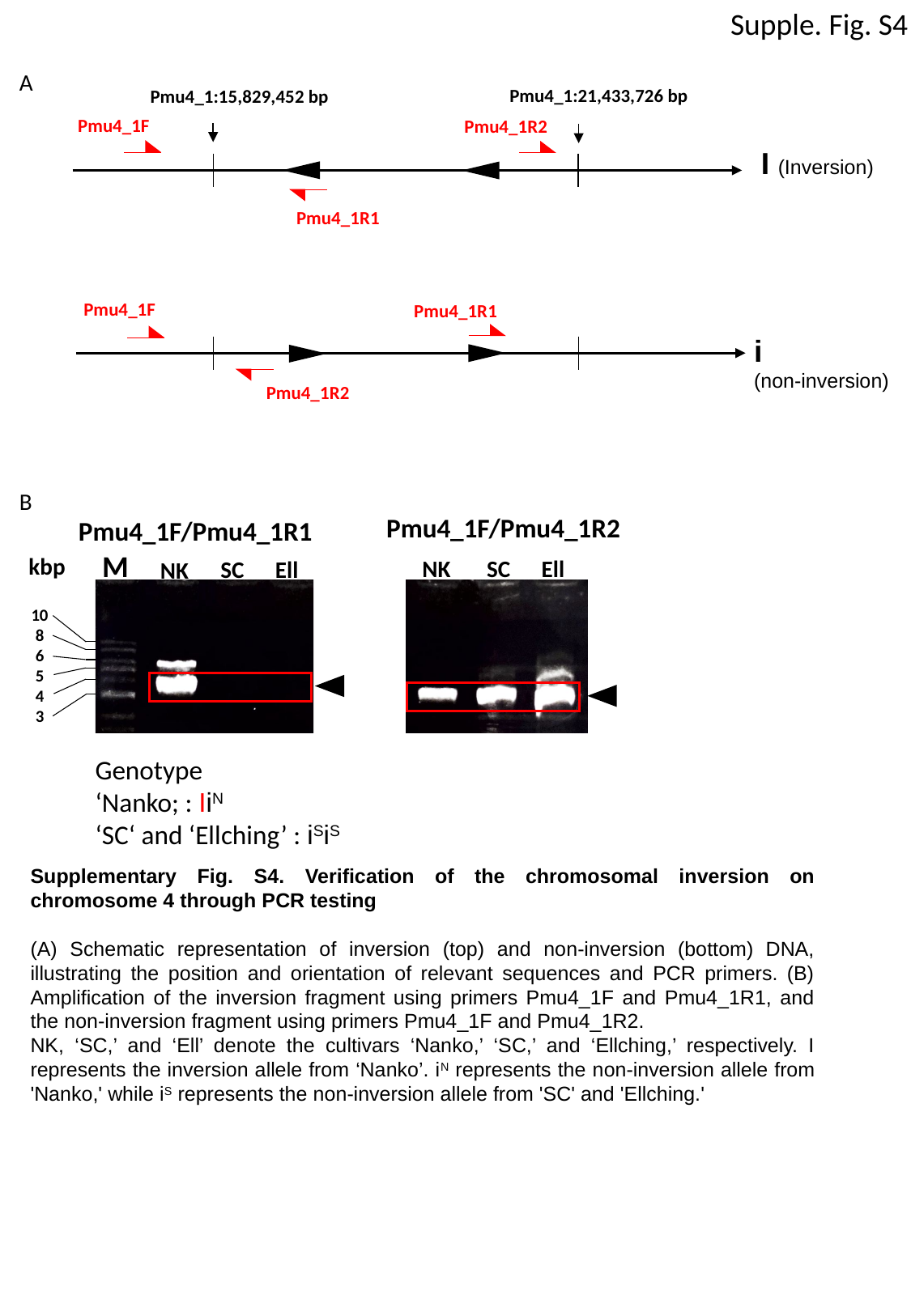

Supple. Fig. S4
A
Pmu4_1:21,433,726 bp
Pmu4_1:15,829,452 bp
Pmu4_1F
Pmu4_1R2
I (Inversion)
Pmu4_1R1
Pmu4_1F
Pmu4_1R1
i
(non-inversion)
Pmu4_1R2
B
Pmu4_1F/Pmu4_1R2
Pmu4_1F/Pmu4_1R1
M
kbp
10
8
6
5
4
3
SC
Ell
NK
SC
Ell
NK
Genotype
‘Nanko; : IiN
‘SC‘ and ‘Ellching’ : iSiS
Supplementary Fig. S4. Verification of the chromosomal inversion on chromosome 4 through PCR testing
(A) Schematic representation of inversion (top) and non-inversion (bottom) DNA, illustrating the position and orientation of relevant sequences and PCR primers. (B) Amplification of the inversion fragment using primers Pmu4_1F and Pmu4_1R1, and the non-inversion fragment using primers Pmu4_1F and Pmu4_1R2.
NK, ‘SC,’ and ‘Ell’ denote the cultivars ‘Nanko,’ ‘SC,’ and ‘Ellching,’ respectively. I represents the inversion allele from ‘Nanko’. iN represents the non-inversion allele from 'Nanko,' while iS represents the non-inversion allele from 'SC' and 'Ellching.'

## Slide 5
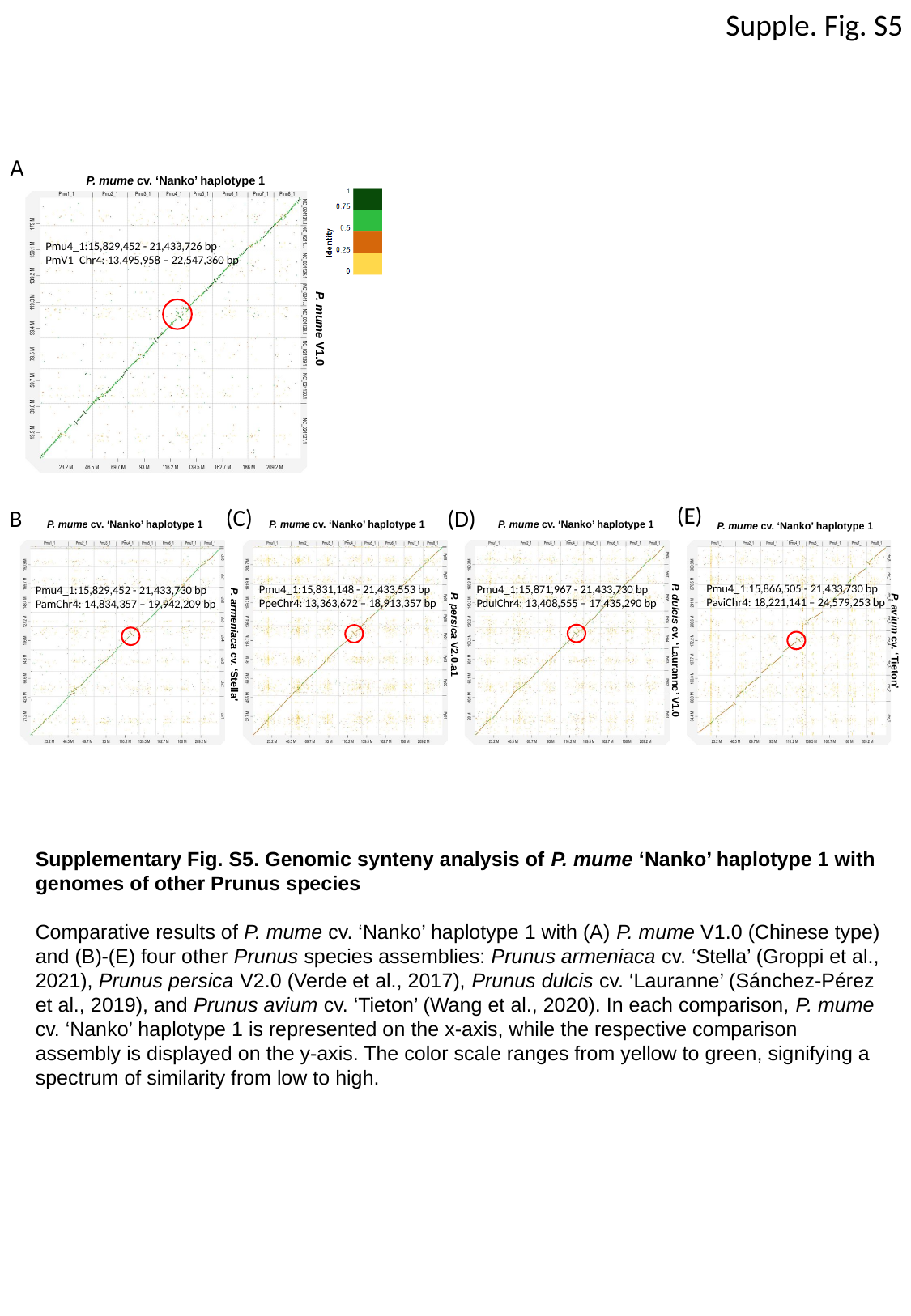

Supple. Fig. S5
A
P. mume cv. ‘Nanko’ haplotype 1
Pmu4_1:15,829,452 - 21,433,726 bp
PmV1_Chr4: 13,495,958 – 22,547,360 bp
P. mume V1.0
(E)
(C)
(D)
B
P. mume cv. ‘Nanko’ haplotype 1
P. mume cv. ‘Nanko’ haplotype 1
P. mume cv. ‘Nanko’ haplotype 1
P. mume cv. ‘Nanko’ haplotype 1
Pmu4_1:15,866,505 - 21,433,730 bp
PaviChr4: 18,221,141 – 24,579,253 bp
Pmu4_1:15,831,148 - 21,433,553 bp
PpeChr4: 13,363,672 – 18,913,357 bp
Pmu4_1:15,871,967 - 21,433,730 bp
PdulChr4: 13,408,555 – 17,435,290 bp
Pmu4_1:15,829,452 - 21,433,730 bp
PamChr4: 14,834,357 – 19,942,209 bp
P. persica V2.0.a1
P. avium cv. ‘Tieton’
P. armeniaca cv. ‘Stella’
P. dulcis cv. ‘Lauranne’ V1.0
Supplementary Fig. S5. Genomic synteny analysis of P. mume ‘Nanko’ haplotype 1 with genomes of other Prunus species
Comparative results of P. mume cv. ‘Nanko’ haplotype 1 with (A) P. mume V1.0 (Chinese type) and (B)-(E) four other Prunus species assemblies: Prunus armeniaca cv. ‘Stella’ (Groppi et al., 2021), Prunus persica V2.0 (Verde et al., 2017), Prunus dulcis cv. ‘Lauranne’ (Sánchez-Pérez et al., 2019), and Prunus avium cv. ‘Tieton’ (Wang et al., 2020). In each comparison, P. mume cv. ‘Nanko’ haplotype 1 is represented on the x-axis, while the respective comparison assembly is displayed on the y-axis. The color scale ranges from yellow to green, signifying a spectrum of similarity from low to high.

## Slide 6
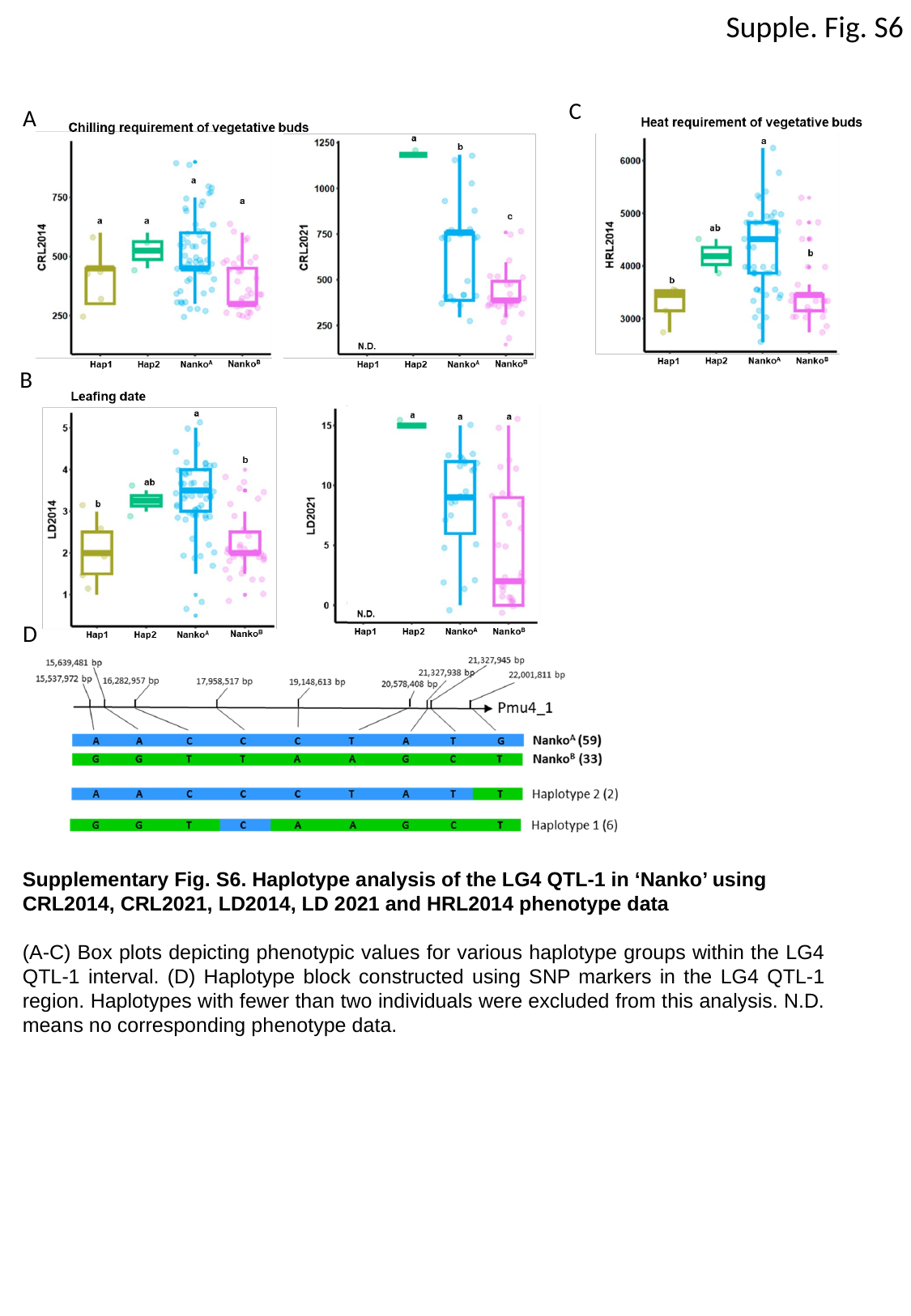

Supple. Fig. S6
C
A
B
D
Supplementary Fig. S6. Haplotype analysis of the LG4 QTL-1 in ‘Nanko’ using CRL2014, CRL2021, LD2014, LD 2021 and HRL2014 phenotype data
(A-C) Box plots depicting phenotypic values for various haplotype groups within the LG4 QTL-1 interval. (D) Haplotype block constructed using SNP markers in the LG4 QTL-1 region. Haplotypes with fewer than two individuals were excluded from this analysis. N.D. means no corresponding phenotype data.

## Slide 7
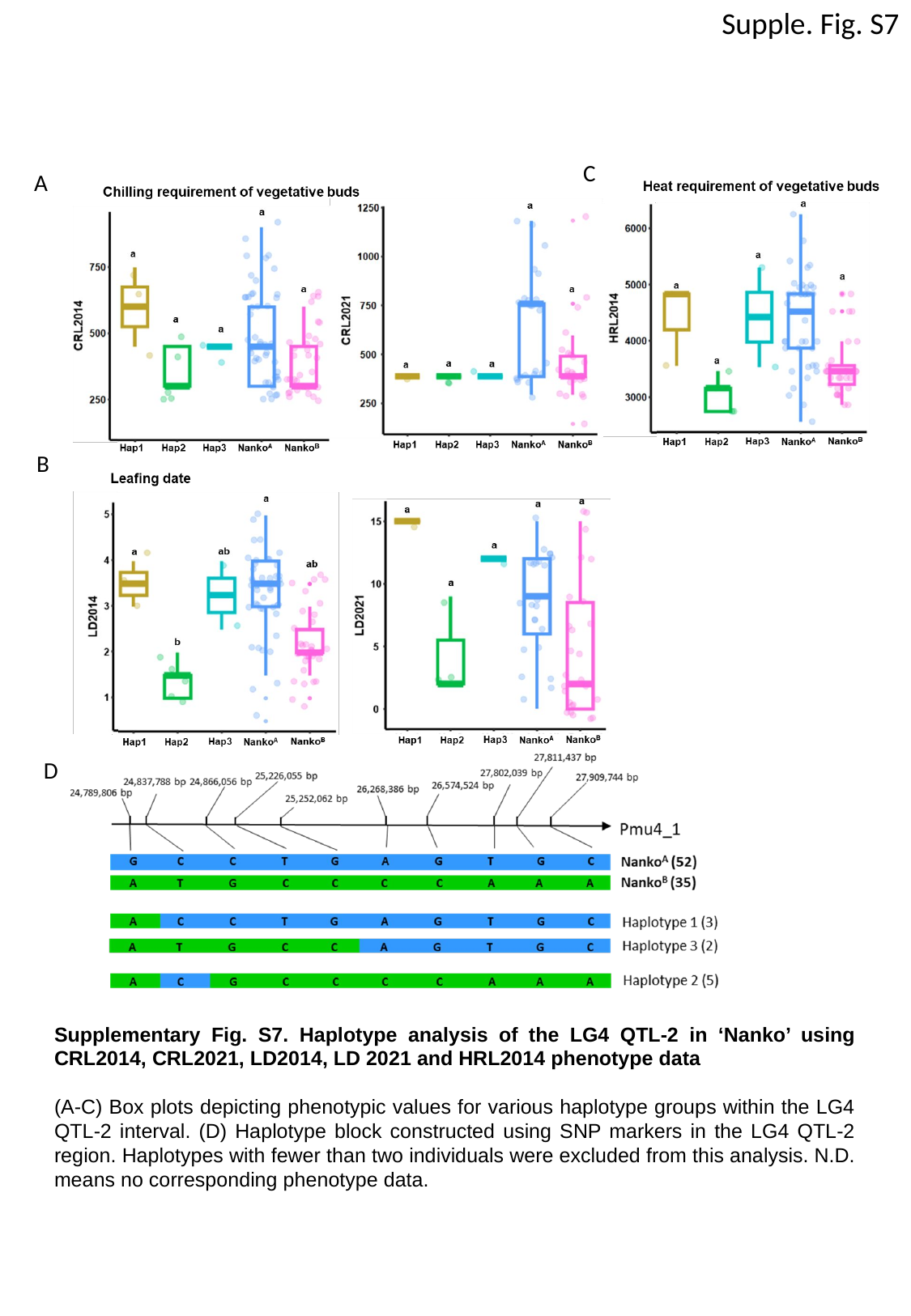

Supple. Fig. S7
C
A
B
D
Supplementary Fig. S7. Haplotype analysis of the LG4 QTL-2 in ‘Nanko’ using CRL2014, CRL2021, LD2014, LD 2021 and HRL2014 phenotype data
(A-C) Box plots depicting phenotypic values for various haplotype groups within the LG4 QTL-2 interval. (D) Haplotype block constructed using SNP markers in the LG4 QTL-2 region. Haplotypes with fewer than two individuals were excluded from this analysis. N.D. means no corresponding phenotype data.

## Slide 8
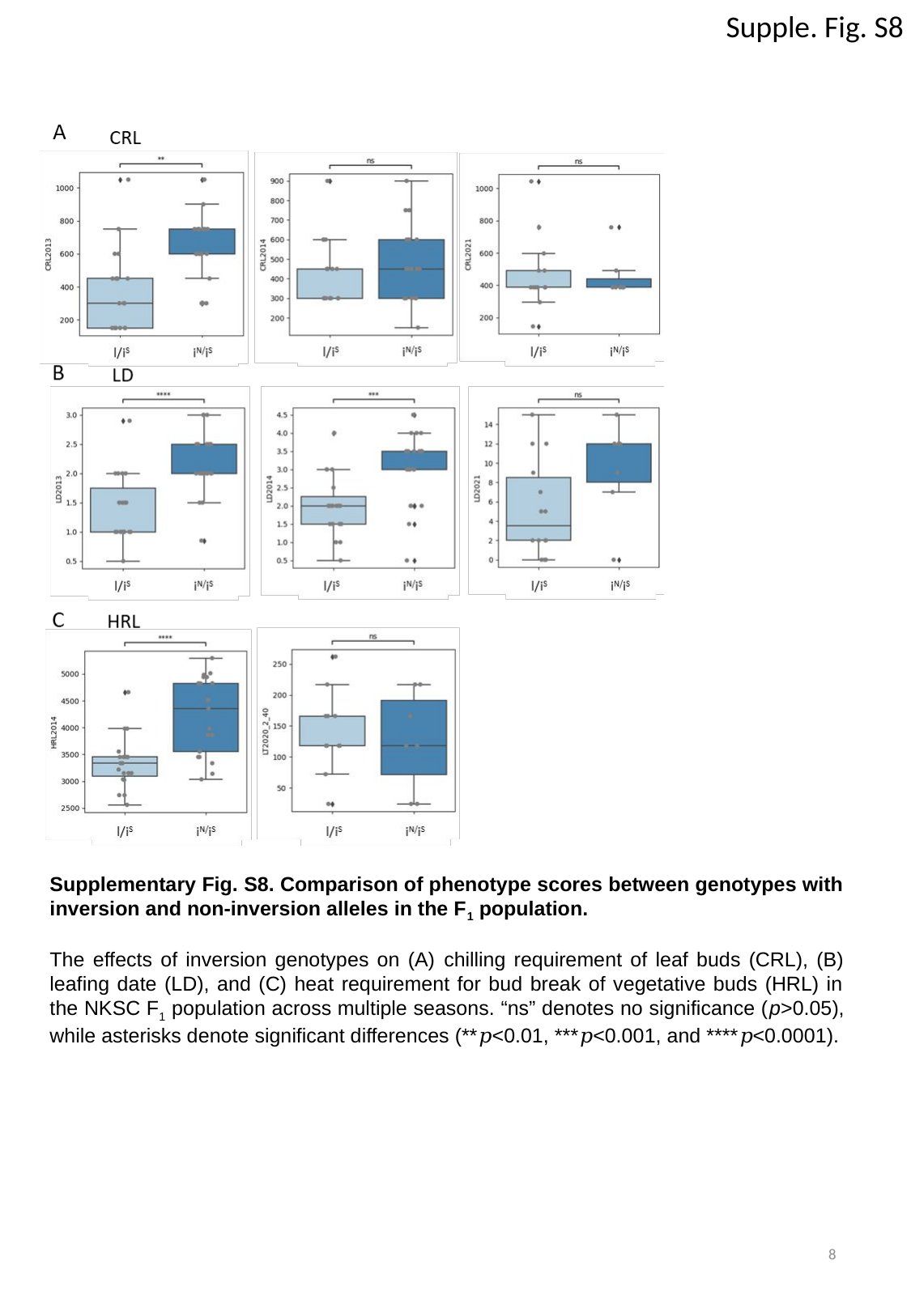

Supple. Fig. S8
Supplementary Fig. S8. Comparison of phenotype scores between genotypes with inversion and non-inversion alleles in the F1 population.
The effects of inversion genotypes on (A) chilling requirement of leaf buds (CRL), (B) leafing date (LD), and (C) heat requirement for bud break of vegetative buds (HRL) in the NKSC F1 population across multiple seasons. “ns” denotes no significance (p>0.05), while asterisks denote significant differences (**𝑝<0.01, ***𝑝<0.001, and ****𝑝<0.0001).
8

## Slide 9
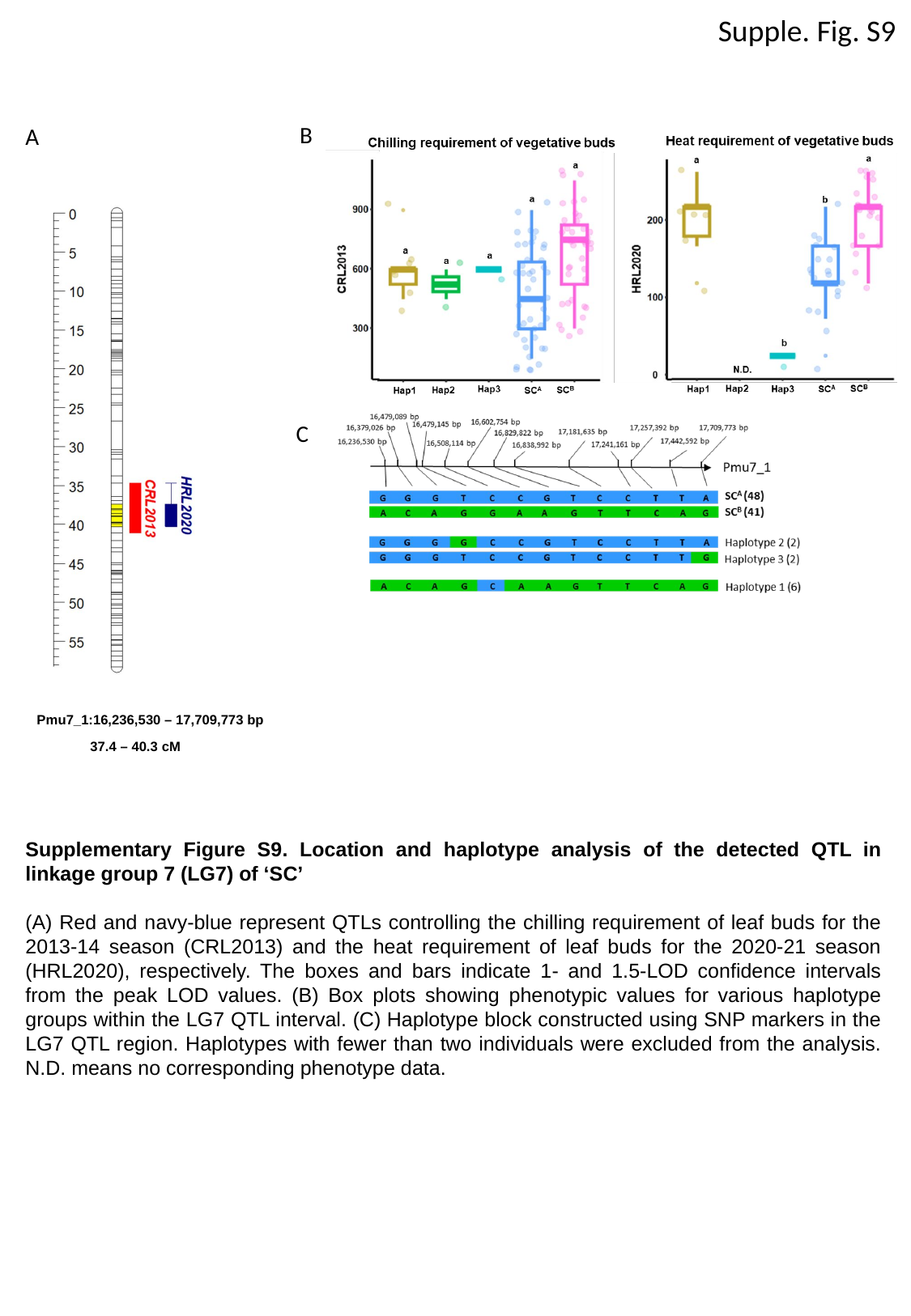

Supple. Fig. S9
B
A
C
Pmu7_1:16,236,530 – 17,709,773 bp
37.4 – 40.3 cM
Supplementary Figure S9. Location and haplotype analysis of the detected QTL in linkage group 7 (LG7) of ‘SC’
(A) Red and navy-blue represent QTLs controlling the chilling requirement of leaf buds for the 2013-14 season (CRL2013) and the heat requirement of leaf buds for the 2020-21 season (HRL2020), respectively. The boxes and bars indicate 1- and 1.5-LOD confidence intervals from the peak LOD values. (B) Box plots showing phenotypic values for various haplotype groups within the LG7 QTL interval. (C) Haplotype block constructed using SNP markers in the LG7 QTL region. Haplotypes with fewer than two individuals were excluded from the analysis. N.D. means no corresponding phenotype data.

## Slide 10
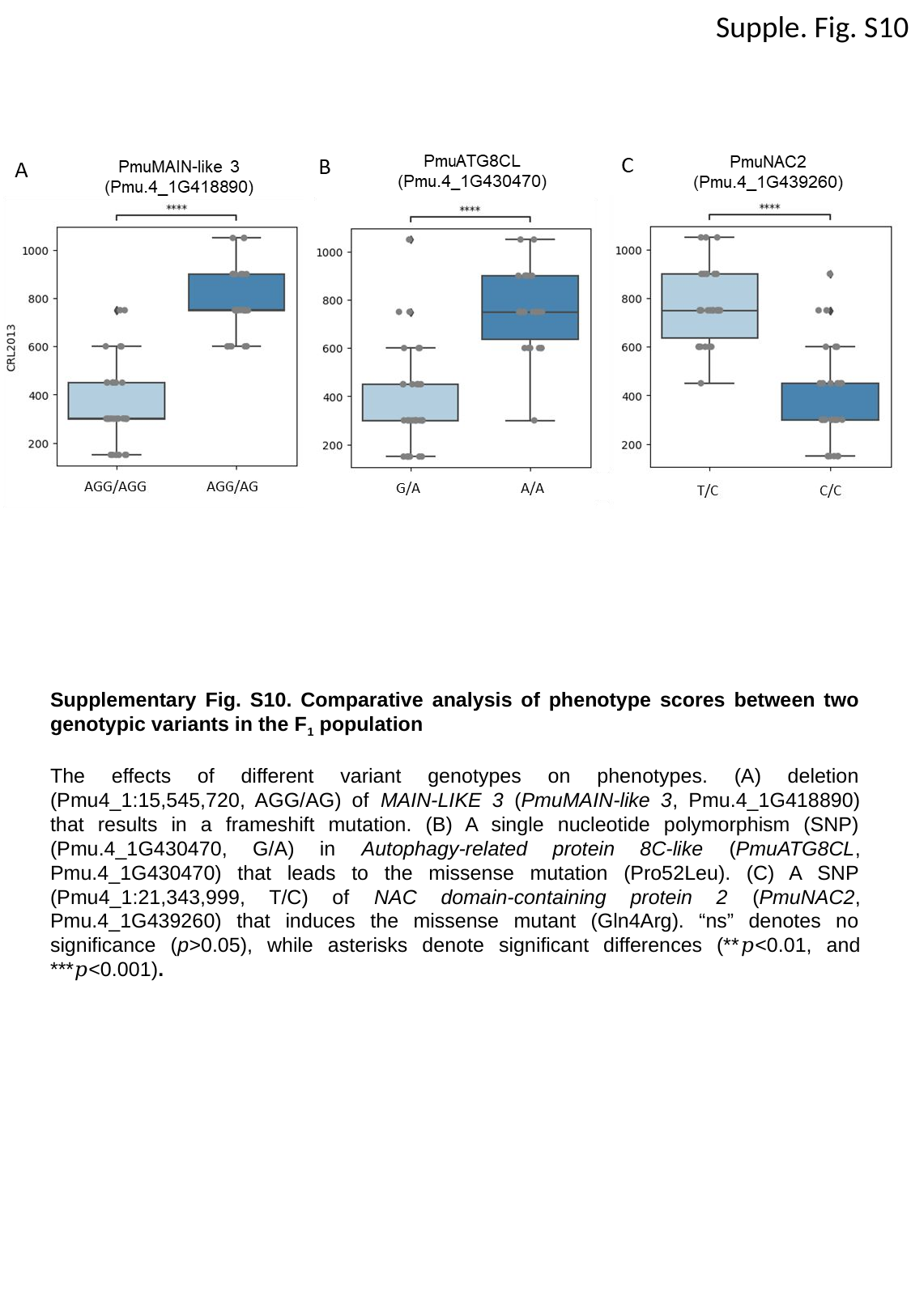

Supple. Fig. S10
Supplementary Fig. S10. Comparative analysis of phenotype scores between two genotypic variants in the F1 population
The effects of different variant genotypes on phenotypes. (A) deletion (Pmu4_1:15,545,720, AGG/AG) of MAIN-LIKE 3 (PmuMAIN-like 3, Pmu.4_1G418890) that results in a frameshift mutation. (B) A single nucleotide polymorphism (SNP) (Pmu.4_1G430470, G/A) in Autophagy-related protein 8C-like (PmuATG8CL, Pmu.4_1G430470) that leads to the missense mutation (Pro52Leu). (C) A SNP (Pmu4_1:21,343,999, T/C) of NAC domain-containing protein 2 (PmuNAC2, Pmu.4_1G439260) that induces the missense mutant (Gln4Arg). “ns” denotes no significance (p>0.05), while asterisks denote significant differences (**𝑝<0.01, and ***𝑝<0.001).
